# Nrf2 overexpression reprograms neural stem cell fate: promoting neuronal differentiation and functional recovery post-ischemic stroke via suppression of the ROS/NF-κB axis

**DOI:** 10.1186/s12967-025-07675-w

**Published:** 2026-01-14

**Authors:** Pengyu Hao, Shihui Liu, Ying Wang, Jiacan Xu, Ruwen Ma, Diqi Mai, Ran Chen, Sijin Tang, Shuaiquan Huo, Renjie Li, Jinyue Gao, Yuting Xiao, Xiaohan Du, Qiushuang Ji, Weijie Zhu, Haixiao Liu, Bodong Wang

**Affiliations:** 1 Department of Neurosurgery, The 960th Hospital of PLA (General Hospital of Jinan Military Command), Jinan, Shandong 250031 China; 2 School of Clinical Medicine, Shandong Second Medical University, Weifang, Shandong 261053 China; 3 School of Clinical Medicine, Jinzhou Medical University, Jinzhou, Liaoning 121001 China; 4 School of Clinical Medicine, Shandong First Medical University, Jinan, Shandong 250117 China; 5 Department of Histology and Embryology, School of Basic Medical Sciences, Cheeloo College of Medicine, Shandong University, Jinan, 250012 China; 6 Department of Neurosurgery, Tangdu Hospital, Fourth Military Medical University, Xi’an, Shaanxi 710038 China

**Keywords:** Cerebral ischaemia, Differentiation, Neural stem cells, Nrf2, Oxidative stress, Proliferation

## Abstract

**Background:**

Neural stem cells (NSCs) have been experimentally used in multiple models and patients, offering great potential for the treatment of neurological disorders such as ischaemic stroke. However, the proliferative and differentiative limitations of NSCs transplants must be overcome to further exploit the clinical potential of NSCs-based therapies.

**Objective:**

This study aimed to elucidate the regulatory role of Nrf2 in NSCs proliferation and neuronal differentiation in vitro, to evaluate the therapeutic potential of transplanting Nrf2-overexpressed NSCs for functional recovery in the MCAO mouse model, and to further explore the synergistic benefits of combining this cell therapy with the pharmacological Nrf2 agonist PTE.

**Methods:**

The study design encompassed in vitro NSC cultures and a mouse MCAO model, involving Nrf2 modulation, transplantation of engineered NSCs, pharmacological activation with PTE, and comprehensive assessment of cellular, molecular, and functional outcomes. Specifically, Nrf2 expression was manipulated using AAV-9. Nrf2-overexpressed NSCs were generated via AAV-9 transduction and stereotaxically transplanted into the lateral ventricle of mice with MCAO, with a subset of these animals additionally receiving the Nrf2 agonist PTE. Nestin, Dcx, BrdU, NeuN, and GFAP expression were evaluated using immunofluorescence; GFAP-positive and NeuN-positive cell proportions were quantified by flow cytometry. Oxidative stress was monitored using DCF fluorescence and MDA ELISA kits. Inflammation was measured with IL-6 and TNF-α ELISA kits. The expression levels of Nrf2, HO-1, NQO1, p65, and NLRP3 were evaluated using western blotting. Neurological deficits were assessed using Clark scores, rotarod test, Morris water maze, grip strength test, cerebral water content measurement, and TTC staining for infarction volume.

**Results:**

Overexpression or knockdown of Nrf2 in vitro (in NSCs) and in vivo (in mouse hippocampus) correspondingly regulated the expression of its downstream effectors, HO-1 and NQO1. In vitro, Nrf2 regulated the proliferation and neuronal differentiation of NSCs. In the MCAO mouse model, Nrf2 ameliorated cerebral oxidative stress and inflammation, and improved functional recovery, as evidenced by improved neurological scores, enhanced performance in behavioural tests, reduced infarction volume, and attenuated cerebral oedema. Transplantation of Nrf2-overexpressed NSCs promoted the proliferation and neuronal differentiation of the grafts, enhanced their neuroprotective effects, and led to superior recovery compared to standard NSCs transplantation. Furthermore, administration of the Nrf2 agonist PTE synergized with Nrf2-overexpressed NSCs transplantation, resulting in augmented anti-inflammatory, antioxidant, and functional benefits.

**Conclusion:**

This study demonstrates that Nrf2 is a key regulator of NSCs proliferation and neuronal fate, both intrinsically and following transplantation. Furthermore, the combination of Nrf2-overexpressed NSCs transplantation and PTE administration synergistically amplified therapeutic outcomes, offering a promising combinatorial strategy to enhance neurorepair after ischemic stroke.

**Supplementary Information:**

The online version contains supplementary material available at 10.1186/s12967-025-07675-w.

## Introduction

Over the past few decades, cerebral ischaemic stroke (CIS) has become increasingly prevalent [1], and remains one of the main causes of morbidity and mortality worldwide [2]. Although intravenous thrombolysis and interventional thrombectomy are currently effective treatments for CIS [1], patients still face a short therapeutic window [1] and the consequences of cognitive and movement impairments [3].

Recently, stem cells have attracted attention owing to their multi-lineage differentiation and immunomodulatory abilities [4]. Neural stem cells (NSCs) are self-renewing populations that are widespread in the subventricular zone and hippocampal dentate gyrus [5]. Studies have suggested that NSCs transplantation is a promising method for ameliorating the pathological symptoms of neurological disorders, especially cerebral ischaemia [6]. However, owing to low rates of NSCs survival and neuronal differentiation, the therapeutic applications of NSCs have been restricted [7, 8]. Recent studies have shown that NSCs are susceptible to senescence-related damage [9]. Generally, for transplantation, NSCs must undergo long-term in vitro culture to obtain sufficient replication, resulting in impaired proliferation and differentiation potential [10]. In addition, the neurological environment under disease conditions induces redox stress sensing and coping mechanisms, including cysteine modification, signalling, metabolic reprogramming, epigenetic alterations, and transcription changes [11]. These may also affect the proliferation and differentiation of NSCs [12]. Therefore, it is important to improve the proliferative and differentiation potentials of NSCs for their future clinical use in stem cell therapy.

Nuclear factor erythroid 2-related factor 2 (Nrf2) is a transcription factor that regulates multiple intracellular antioxidant enzymes [13]. Studies have shown that Nrf2 plays antioxidant and anti-inflammatory roles in many neurological disorders [13]. Recent studies have suggested that Nrf2 promotes the survival and neurogenesis of NSCs in neurodegenerative diseases [14] and mitigates the decline in NSCs function during aging [15]. However, whether Nrf2 can regulate the fate of endogenous or transplanted NSCs and thus affect the outcome of CIS remains unknown. Therefore, elucidating the role of Nrf2 in endo- and exogenous NSCs may contribute to their therapeutic potential.

Although differences in tissue source (embryonic vs. adult) and microenvironment (healthy vs. diseased) exist, the core signalling pathways governing NSCs self-renewal, differentiation, and survival are highly conserved between the developing and adult brain. This conservation is evidenced by numerous seminal studies on adult neurogenesis and post-ischemic repair, which have successfully utilized embryonic-derived NSCs or cell lines to identify key molecules with congruent functions in vivo [16, 17]. Therefore, to investigate the fundamental mechanisms regulating NSCs fate, embryonic-derived NSCs were utilized for the in vitro arm of this study.

In the present study, we investigated the effects of Nrf2 on NSCs proliferation and differentiation in vitro and in a middle cerebral artery occlusion (MCAO) mouse model, as well as its effects on redox stress and inflammation in the brain. Next, we established Nrf2-overexpressed NSCs to reveal the effects of Nrf2 on NSCs transplantation after MCAO and to explore the effects of Nrf2-overexpressed NSCs and PTE combined Nrf2-overexpressed NSCs transplantation therapy on MCAO prognosis.

## Material and methods

### Culture of neural stem cells

The NSCs used in vitro studies were isolated from the hippocampus of C57BL/6 embryos at day 12.5 post coitus and were expanded in culture medium containing all essential supplements for mouse NSCs propagation, ensuring their canonical properties. C57BL/6 mouse NSCs and NSCs culture medium were purchased from Cyagen Biosciences, Inc. (Guangzhou, China). The NSCs were incubated in a humidified atmosphere of 5% CO_2_ at 37 °C, with media changes every 2 days. In our study, all experiments were performed using neural stem cells between passages 3 and 5. This low-passage range is well-established in the literature to maintain the stemness, proliferation capacity, and multi-lineage differentiation potential of NSCs without signs of senescence [18–20].

### Infection of adeno-associated virus vectors

AAV-9 was purchased from Cyagen Biosciences Inc. (Guangzhou, China), encoding the gene *eGFP*, mouse *Nfe2l2* (gene name of Nrf2), empty plasmid, shRNA against mouse *Nfe2l2*, or scrambled shRNA, with a Cytomegalovirus or U6 promoter. The vendor provided functional validation data for their viral products, and we selected a sequence with confirmed high efficiency. The sequence of mouse shRNA targeting *Nfe2l2* was 5’-CCCGA ATTACA GTGTCT TAATCT CGAGAT TAAGAC ACTGTA ATTCGG G-3’, and the sequence of scrambled shRNA was 5’-CCTAA GGTTAA GTCGCC CTCGCT CGAGCG AGGGCG ACTTAA CCTTAG G-3’. In vitro, NSCs were transfected with the vectors at a MOI of 10,000:1, and 48 h later, the transfection mixture was replaced with complete medium, and green fluorescence was observed under a fluorescence microscope to ensure the completion of transfection.

### Animal and in vivo experimental design

Male C57BL/6 mice (6–8 weeks old) were provided by the Laboratory Animal Centre of Shandong University. All animals were fed ad libitum and kept under a normal 12 h light/12 h dark cycle. .

Experiment 1: Forty-eight mice were randomly divided into 4 groups, with 12 mice in each group: Sham, MCAO, MCAO + shNrf2, and MCAO + Nrf2. First, MCAO or a sham operation was performed as previously described [21]. Subsequently, the mice in each group received stereotaxic injections of 0.5 µl AAV-9 (titre > 1.0 × 10^12^ GC/ml) in the hippocampal area (AP: -1.5 to -3.0; ML: ±1.5; DV: -1.5 to -2.5; Bregma was the origin of coordinates. Unit: mm). These AAVs carried either scrambled shRNA, Nrf2 target shRNA, or the Nrf2 overexpression plasmid.

Experiment 2: Nrf2-overexpressed NSCs were generated by transducing NSCs with AAV-9 as described above. Forty-eight mice were randomly divided into 4 groups, with 12 mice in each group: Sham, MCAO, MCAO + NSCs, and MCAO + AAV-Nrf2-NSCs. First, MCAO or a sham operation was performed as described above. Subsequently, the mice in the MCAO + NSCs and MCAO + AAV-Nrf2-NSCs groups received stereotaxic injections of 4 µl NSCs (1.0 × 10^6^ cells) and 4 µl Nrf2-overexpressed NSCs (1.0 × 10^6^ cells), respectively, in the right lateral ventricle (AP: -0.3 to -0.9; ML: +1.0; DV: -2.0 to -2.5; Bregma was the origin of coordinates. Unit: mm). Simultaneously, mice in the Sham and MCAO groups were injected with the same amount of normal saline.

As previously mentioned, Nrf2 or control genes were transduced into NSCs in vitro using eGFP-labelled AAV-9. Successful transduction was confirmed by the presence of green fluorescence observed under a fluorescence microscope prior to transplantation. This eGFP expression allowed for the clear identification of donor transplanted cells (eGFP-positive) versus host cells (eGFP-negative) in subsequent histological analyses.

Mice were injected with PTE intraperitoneally: A subset of mice that received Nrf2-overexpressed NSCs were further administered the Nrf2 agonist PTE. In accordance with previous studies, PTE was dissolved in 10% DMSO and 90% saline (with 1% Tween 80 added) and administered via intraperitoneal injection immediately after MCAO (PTE, 10 mg/kg; vehicle, saline mixture solution) [22].

### BrdU staining

NSCs were cultured with 20 mM BrdU for 72 h, centrifuged, and dried on lysine-coated slides. Next, the cells were fixed with 4% paraformaldehyde for 30 min, incubated with 2 M HCL for 60 min, and incubated with 0.1 M sodium borate for 10 min at 25 °C. Finally, the cells were incubated with anti-BrdU primary antibody (1:100; Wanleibio, Shenyang, China) for 60 min at room temperature and Alexa Fluor 555 conjugated Donkey anti-rabbit secondary antibody (1:500; Beyotime Biotechnology, Shanghai, China) for 60 min at 25 °C in the dark. Nuclei were stained with DAPI (1:200, Sangon Biotech, Shanghai, China) for 20 min. Images were acquired using a laser confocal scanning microscope (Nikon A1R, Tokyo, Japen).

### Immunofluorescence staining

The cells were centrifuged, dried, and fixed on lysine-coated slides, and frozen brain tissue sections were prepared as previously described [23]. The cells and sections were permeabilised with 0.3% Triton X-100 for 15 min and blocked with 5% BSA for 30 min. The slides were incubated with primary antibodies overnight at 4 °C. The primary antibodies used were as follows: rabbit anti-mouse NeuN (1:300, Abcam, Boston, MA, USA), GFAP (1:200, Wanleibio), Nestin (1:200, Abclonal, Woburn, MA, USA), and Dcx (1:200, Abclonal). The secondary antibody Alexa Fluor 555 conjugated Donkey anti-rabbit (1:500; Beyotime Biotechnology), was incubated for 60 min at room temperature in the dark. Nuclei were stained with DAPI (1:200, Sangon Biotech) for 20 min. Images were acquired using a laser confocal scanning microscope (Nikon A1R).

### Dichlorofluorescein staining

The DCF staining was performed as previously reported [24], and DCF-positive cells were counted using ImageJ software (version 1.46r, Wayne Rasband, National Institutes of Health, USA).

### Measurement of MDA, IL-6, and TNF-α

MDA, IL-6, and TNF-α levels were measured using an MDA assay kit (Beyotime Biotechnology), and TNF-α and IL-6 ELISA kits (Sangon Biotech), as previously reported [25].

### Western blotting

Whole-cell protein samples were prepared, separated, and transferred to PVDF membranes as previously described [25]. Membranes were blocked with 5% BSA, and incubated with primary antibodies, including rabbit anti-mouse Nestin (1:1000, Proteintech), Dcx (1:2000, Proteintech), Nrf2 (1:1000, Abclonal), NQO1 (1:1000, Wanleibio), HO-1 (1:1000, Wanleibio), p65 (1:1000, Proteintech), NLRP3 (1:1000, Abclonal) and β-actin (1:2000, Wanleibio). Horseradish peroxidase-labelled goat anti-rabbit secondary antibodies were purchased from Beyotime Biotechnology. Finally, the bands were visualised using an ultrasensitive ECL chemiluminescence kit (Beyotime Biotechnology) and analysed using the ImageJ software (version 1.54p).

### Flow cytometry

Neural stem cell differentiation fates were quantified 72 h post-intervention using flow cytometry (BD FACSymphony A5). Fixed and permeabilized cells were stained with Alexa Fluor 488-conjugated anti-GFAP and PE-conjugated anti-NeuN, with isotype controls defining gating thresholds [26]. In short, a sequential gating strategy was employed to accurately quantify the differentiation fate of transfected neural stem cells. Initially, the main cell population was gated on a forward scatter-area (FSC-A) versus side scatter-area (SSC-A) dot plot to exclude small debris based on cell size and granularity. Subsequently, single cells were discriminated and selected using a forward scatter-height versus forward scatter-area (FSC-H vs. FSC-A) plot to exclude cell doublets. To exclude dead cells, the cells were stained with PI and the live cell population was gated as PI-negative. Appropriate isotype controls were used for all antibodies to calibrate background fluorescence. GFAP-positive/NeuN-negative populations indicated astrocytic differentiation, while GFAP-negative/NeuN-positive populations denoted neuronal commitment. Data from three biological replicates (> 10,000 events/sample) were analysed blindly in FlowJo v10.9.

### Rotarod test

Motor coordination and endurance were evaluated using an accelerated Rotarod test. Mice were habituated to the apparatus through daily 5-min stationary exposure for 2 consecutive days prior to formal testing. During testing sessions, each mouse was placed on the rotating rod facing against the direction of rotation to eliminate directional bias. The rod acceleration protocol was set from 4 rpm to 20 rpm over a 5-min (300 s) trial, with latency to fall automatically recorded by infrared sensors. Mice remaining on the rod for the full duration were assigned a maximum latency of 300 s. Three independent trials per mouse were conducted with 30-min rest intervals between trials to prevent fatigue-induced performance decline. The outcome measure was the mean latency to fall (seconds), calculated from three trials.

### Limb grip strength test

Forelimb and hindlimb strength were quantified using a digital grip strength dynamometer. Prior to testing, the device was calibrated following manufacturer specifications and zero-adjusted to ensure measurement accuracy. To minimize stress-induced performance variability, mice were acclimatized to the testing room for 30 min under dim light with white noise masking. During testing, each mouse was gently held by the base of the tail and lowered toward the horizontal traction grid until the limbs grasped the grid spontaneously. The mouse was then pulled backward at a constant velocity along the axis parallel to the grid until grip release occurred. The peak force (g) was automatically recorded by the dynamometer’s force transducer. Three consecutive trials were performed with 5-min inter-trial intervals to prevent fatigue, and the mean value across trials was used for analysis. The outcome measure was the peak grip force (grams), averaged across three consecutive trials.

### Morris water maze test

Spatial learning and memory were assessed using a Morris water maze (MWM) system beginning on day 3 post-MCAO [27]. The circular pool (120 cm diameter, 50 cm height) was filled with opaque water (22 ± 1 °C) rendered non-transparent by white food-safe dye. During the visible platform phase (days 3–5), mice underwent four daily trials (60 s/trial, ≥ 15-min inter-trial intervals) to locate a cued platform (10 cm diameter) positioned 1 cm above water level, with entry points randomized across four quadrants. This was followed by a hidden platform phase (days 6–8) where the platform was submerged 1 cm below the surface, requiring navigation using distal spatial cues. On day 9, a motor navigation test with the hidden platform controlled for swim capacity confounds. A probe trial on day 10 removed the platform to measure spatial memory retention over 60 s, quantifying time in target quadrant and platform crossings (within a 10-cm virtual zone). An automated video tracking system recorded escape latency, trajectory metrics and other data. Mice failing to locate the platform within 60 s were guided to it and allowed 30 s of spatial reinforcement. The primary outcome measures were escape latency (seconds) during the hidden platform trials and the time spent in the target quadrant (seconds) during the probe trial.

### Brain water content measurement

Cerebral oedema was quantified via the wet-dry weight method at designated post-MCAO time points [28]. Mice were anesthetized and rapidly decapitated. Brains were extracted within 60 s and placed on a pre-chilled plate (0–4 °C). Meninges and surface vessels were meticulously removed, followed by gentle blotting on filter paper to eliminate residual moisture. Wet weight (WW) of cortical tissues was immediately measured using a precision microbalance. Samples were desiccated at 110 °C for 24 h until constant mass, and dry weight (DW) was recorded. Water content was calculated as: Water content (%) = (WW - DW/WW) × 100. Triplicate measurements per sample ensured technical reproducibility. The result was expressed as brain water content (percentage).

### TTC staining

Cerebral infarction was assessed using TTC staining [28]. Mice were euthanized at designated post-MCAO time points, and brains were rapidly extracted. Six consecutive 1-mm coronal sections were cut, incubated in 2% TTC solution (37 °C, 15 min), and fixed in 4% paraformaldehyde. Digital images were acquired by photography under standardized lighting conditions. Infarct area was quantified using ImageJ software (version 1.54p), and the total infarct volume was calculated and expressed as a percentage of the total area of the entire coronal brain sections.

### Neurological deficit assessment (Clark score)

Neurological deficits were evaluated at designated time points after MCAO surgery using the modified Clark scoring system. Assessments were performed by an investigator blinded to the experimental groups. The scoring system evaluates general status, motor function, and reflex responses, with a total possible score ranging from 0 (no deficit) to 28 (maximum deficit). The outcome measure was the neurological deficit score, with a higher score indicating more severe neurological impairment.

### Single-cell RNA sequencing analysis

To investigate the molecular events occurring in neural stem cells following mouse MCAO, public single-cell RNA sequencing data (GSE174574) were analysed using Seurat. After quality control and t-SNE clustering, cell clusters were manually annotated by referencing canonical marker genes from established scRNA-seq literature and databases. NSCs identified during annotation underwent sub-clustering. Differentially expressed genes in NSCs subpopulations were identified (Wilcoxon test, *P* < 0.05). GO and KEGG enrichment analyses were performed using clusterProfiler v4.0 with significance threshold of *P* < 0.05.

### Statistical analysis

All data were analysed using GraphPad Prism 10 (GraphPad Software Inc., CA, USA) and are shown as mean ± standard deviation. Differences between groups were compared using one-way analysis of variance, and differences between two groups were compared using the unpaired Student’s t-test. Statistical significance was set at *P* < 0.05.

### Ethical approval

All animal experiments were approved by the Ethics Committee of the 960th Hospital (Approval No. 2018-08) and were conducted in accordance with the NIH Guide for the Care and Use of Laboratory Animals. Mice were housed under standard conditions with ad libitum access to food and water. All efforts were made to minimize suffering; surgical procedures were performed under anaesthesia, and postoperative analgesia was provided. Humane endpoints were strictly observed. At the conclusion of the study, euthanized animals and all biological tissues were disposed of in a hygienic and ethical manner via authorized medical waste incineration. This study is reported in compliance with the ARRIVE guidelines 2.0.

## Results

### Altered NSCs States in MCAO mice revealed by single-cell sequencing

**Fig. 1:**
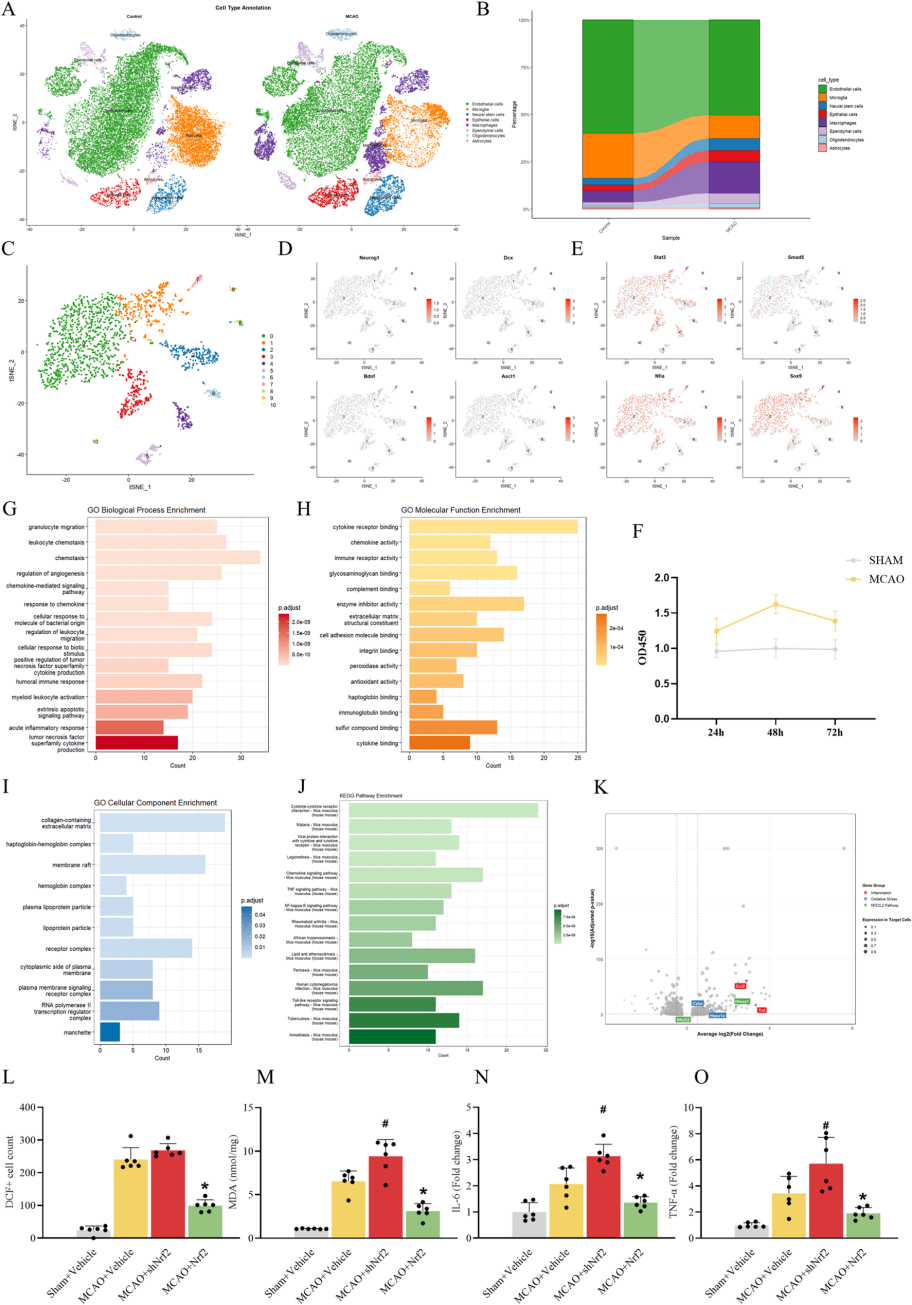
scRNA-seq reveals altered NSC states and Nrf2’s role in ischemia. (**A**) t-SNE of 49,142 dissociated cells from three MCAO mouse and three control individuals based on six single-cell sequencing datasets from the GEO database, showing major cell types identified through integrated analysis. (**B**) Proportional clustering of astrocytes comparing the MCAO model and control groups. (**C**) t-SNE visualization of neural stem cell subclusters identified through secondary subclustering analysis in the MCAO model. Feature plot verifying clustering assignments by representative cell-specific gene expression, (**D**) Stat3, Smad5, Nfia, and Sox9, (**E**) Neurog1, Dcx, Bdnf, and Ascl1. (**F**) CCK-8 assay showing the proliferative capacity of neural stem cells from SHAM and MCAO groups at 24, 48, and 72 h, as measured by OD450 values. Bar plots showing enrichment of GO pathways (**G**-**I**) and enrichment of KEGG pathways(J) in neural stem cells based on differentially expressed genes (DEGs) identified in MCAO vs. SHAM.(K)Volcano plot displaying significantly upregulated (right side) and downregulated (left side) genes in neural stem cells, with key marker genes (Ccl2, Tnf, Cyba, Hspa1a, and Hmox1) labelled.(**L**, **M**) Oxidative level was valued by DCF staining and MDA assay. The histograms showed the number of DCF-positive cells and MDA levels. (**N**, **O**) The histograms showed the levels of inflammatory factor IL-6 and TNF-α, measured with ELISA kits. The data are expressed as means ± SD (*n* = 6). ^*^P < 0.05 vs. MCAO + Vehicle group, ^#^*P* < 0.05 vs. MCAO + Vehicle group

**Fig. 2:**
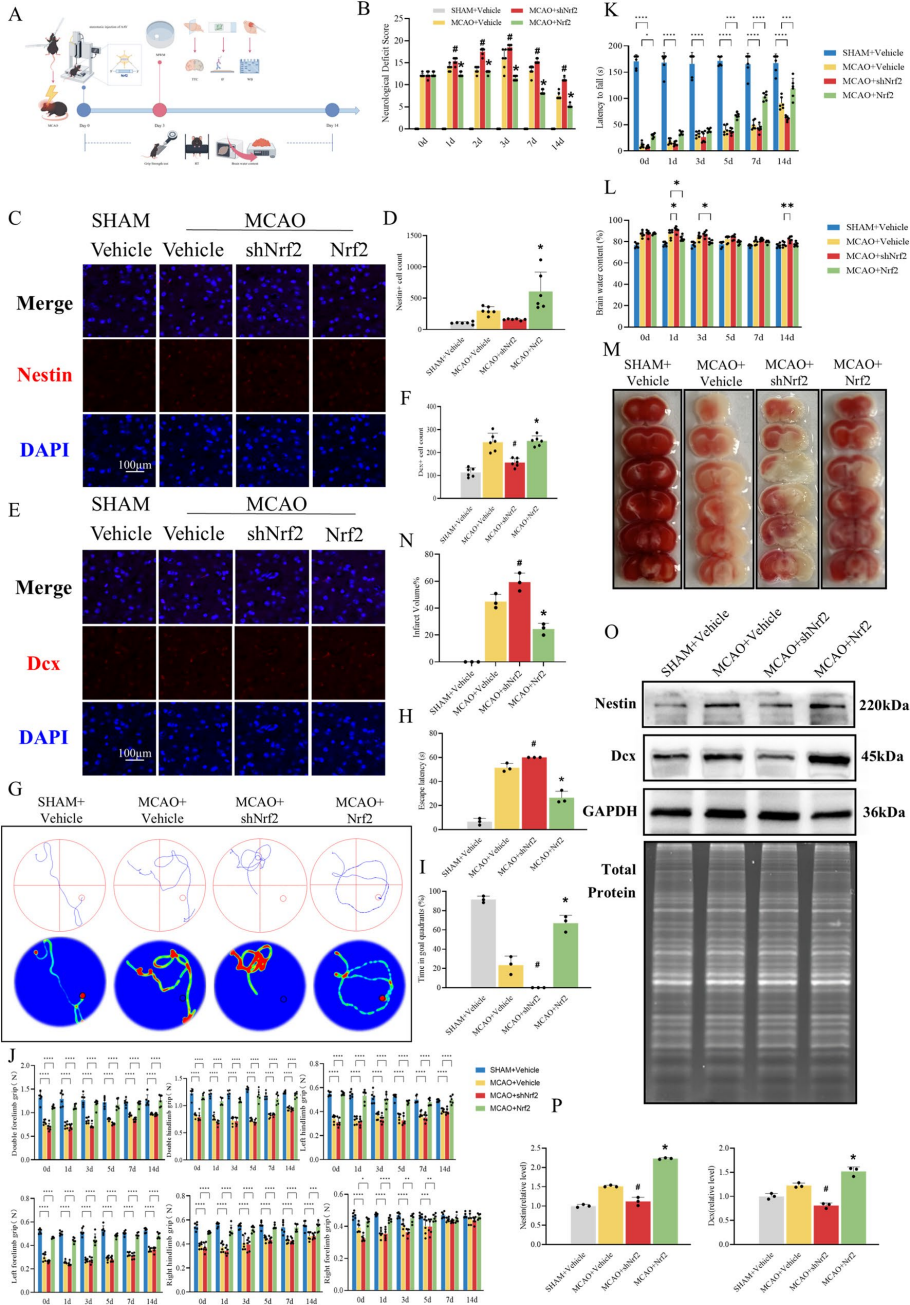
Nrf2 enhances endogenous neurogenesis and neurological functional recovery. (**A**) The experimental protocol of the role of Nrf2 overexpression in neural stem cell differentiation in the MCAO mouse model. (**B**) Bar chart showing neurological deficit scores of each group from day 0 to day 14 after MCAO. The data are expressed as means ± SD (*n* = 6). ^*^P < 0.05 vs. MCAO + Vehicle group, ^#^*P* < 0.05 vs. MCAO + Vehicle group. (**C**-**F**) Neuronal fate shift potential of endogenous NSCs was evaluated by Nestin and Dcx immunofluorescence. The histograms showed the percentage of Nestin- and Dcx-positive cells among 4 groups. The data are expressed as means ± SD (*n* = 6). ^*^P < 0.05 vs. MCAO + Vehicle group, ^#^*P* < 0.05 vs. MCAO + Vehicle group. (**G**-**I**) Representative swimming trajectories of MWM test, bar chart showing escape latency and time spent in the target quadrant. The data are expressed as means ± SD (*n* = 3). ^*^P < 0.05 vs. MCAO + Vehicle group, ^#^*P* < 0.05 vs. MCAO + Vehicle group. (**J**) Histogram displaying grip strength test results across four experimental groups. The data are expressed as means ± SD (*n* = 6). ^*^P < 0.05, ^**^P < 0.01, ^***^P < 0.001, ^****^P < 0.0001. (**K**) Histogram showing the latency to fall in the rotarod test across four experimental groups. The data are expressed as means ± SD (*n* = 6). ^*^P < 0.05, ^**^P < 0.01, ^***^P < 0.001, ^****^P < 0.0001. (**L**) Histogram quantifying brain water content (%) across four experimental groups. The data are expressed as means ± SD (*n* = 6). ^*^P < 0.05, ^**^P < 0.01, ^***^P < 0.001, ^****^P < 0.0001. (**M**, **N**) Representative TTC staining and quantitative analysis of the infarct volume in each group. The data are expressed as means ± SD (*n* = 3). ^*^P < 0.05 vs. MCAO MCAO + Vehicle group, ^#^*P* < 0.05 vs. MCAO + Vehicle group. (**O**, **P**) Western blot analysis of Nestin and Dcx expression in brain tissues of each group of mice. The data are expressed as means ± SD (*n* = 3). ^*^P < 0.05 vs. MCAO + Vehicle group, ^#^*P* < 0.05 vs. MCAO + Vehicle group

Analysis of six single-cell sequencing datasets from the GEO database [29] revealed distinct cellular compositions in MCAO brain tissues. t-SNE of 49,142 dissociated cells identified 16 distinct clusters across the experimental groups. Cell-type annotation revealed the following clusters: endothelial cells, microglia, neural stem cells, epithelial cells, macrophages, ependymal cells, oligodendrocytes, and astrocytes (Fig. 1A) [30]. Cluster Neural stem cells showed expansion after MCAO (Fig. 1B). The CCK-8 proliferation assay demonstrated that neural stem cells from MCAO mice exhibited significantly enhanced proliferative capacity compared to the control group (Fig. 1F) [16]. Subsequently, we performed re-clustering on the neural stem cell population and identified 11 distinct subclusters (Fig. 1C). Combined with differential gene expression and pathway enrichment analyses, our results demonstrated that NSCs after MCAO exhibited a transcriptional shift characterized by upregulated expression of Stat3, Smad5, Nfia, and Sox9 [31], which are associated with glial lineage commitment, while the levels of key neuronal differentiation regulators such as Neurog1, Dcx, Bdnf, and Ascl1 remained largely unaltered (Fig. 1D and E) [32–34]. Together, these transcriptional changes revealed a potential bias in NSCs differentiation potential post-ischemia. Subsequent analysis focused on the broader transcriptional response. These NSCs displayed marked enrichment of gene sets related to inflammatory responses, oxidative stress, and antioxidant activity (Fig. 1G, H and I) [35]. KEGG analysis revealed significant enrichment of neuroinflammatory pathways, including the TNF signalling pathway and NF-κB signalling pathway (Fig. 1J) [17]. Notably, upregulated expression of Ccl2, Tnf, Cyba, Hspa1a, and Hmox1 was observed (Fig. 1K) [36]. Based on these transcriptional alterations indicating inflammatory and oxidative stress responses, we next investigated whether modulating Nrf2 could influence this pathophysiological state. AAV-9, which interferes with or overexpresses Nrf2, was injected into the hippocampal area of MCAO mice using a stereotaxic instrument (Fig. 2A), and brain tissues were obtained after 7 days for subsequent analyses. Subsequently, brain sections were stained with DCFH-DA, revealing a significant increase in DCF-positive cells after MCAO [24]. AAV-9-mediated Nrf2 overexpression markedly reduced DCF-positive cells in MCAO brains (Fig. 1L). Fresh hippocampal tissues were harvested from each group, and MDA, IL-6, and TNF-α levels were quantified using ELISA kits. Results demonstrated that MCAO elevated hippocampal MDA, IL-6, and TNF-α levels [25]. These biomarkers were further increased following Nrf2 knockdown but decreased after Nrf2 overexpression (Fig. 1M-O).

### Effects of Nrf2 modulation on endogenous neurogenesis and functional outcomes after MCAO

AAV-9-mediated Nrf2 knockdown or overexpression was performed as described previously. Behavioural assessments, histological analyses, and other endpoint assays were conducted at designated time points as illustrated in the experimental timeline (Fig. 2A). Nestin and DAPI were immunofluorescently labelled. The results showed that the number of Nestin-positive cells increased after MCAO [37] and decreased after Nrf2 knockdown; however, the difference was not significant. Compared to the MCAO group, the number of Nestin-positive cells significantly increased after Nrf2 overexpression (Fig. 2C and D). Dcx and DAPI staining were also performed. It was found that the number of Dcx-positive cells in MCAO mice increased [37], and interference with Nrf2 impaired the increase in Dcx-positive cells (Fig. 2E and F). To further validate the regulatory role of Nrf2 in NSCs differentiation, we examined Nestin and Dcx expression levels by Western blotting. Results revealed that Nrf2 overexpression significantly upregulated both Nestin and Dcx protein levels, consistent with immunofluorescence findings (Fig. 2O) [38, 39]. Collectively, these findings suggest that Nrf2 modulates neuronal fate shift in endogenous NSCs within MCAO brains. Next, an assessment of neurological deficits with Clark scores revealed that the interference of Nrf2 exacerbated the neurological deficits in MCAO mice, with a significant increase in the neurological deficit score, while overexpression of Nrf2 significantly ameliorated the neurological deficits in MCAO mice, accompanied by a significant decrease in the neurological deficit score (Fig. 2B) [40]. Concurrently, behavioural assessments corroborated these findings. The rotarod test demonstrated that Nrf2 knockdown accelerated the fall latency in MCAO mice, whereas Nrf2 overexpression significantly prolonged the latency to fall (Fig. 2K) [40]. Grip strength test revealed weakened forelimb/hindlimb strength in Nrf2-knockdown MCAO mice, which was ameliorated by Nrf2 overexpression (Fig. 2J). Notably, ipsilateral forelimb weakness was observed in MCAO mice but showed gradual recovery over time. Morris water maze data further complemented cognitive evaluation: Nrf2 knockdown prolonged escape latency and reduced target quadrant duration in MCAO mice, while Nrf2 overexpression markedly rescued these spatial memory deficits (Fig. 2G-I) [41]. In summary, Nrf2 overexpression ameliorates motor, cognitive, and memory deficits induced by MCAO. Subsequently, cerebral water content was measured at specified time points. Results demonstrated a significant elevation in brain water content during the early phase post-MCAO (days 1 and 3). Nrf2 overexpression effectively ameliorated this cerebral oedema. Conversely, Nrf2 knockdown not only exacerbated early-stage oedema but also induced persistent aggravation of brain water content at day 14 (Fig. 2L) [42]. TTC staining was employed to evaluate the neuroprotective effect of Nrf2 overexpression on cerebral infarction volume. Quantitative analysis revealed a significantly higher infarction volume in the Nrf2-knockdown group (59.29% ± 6.67%) compared to the MCAO group (44.89% ± 5.34%). Conversely, the Nrf2-overexpression group exhibited a markedly reduced infarction volume (24.47% ± 4.18%) (Fig. 2M and N) [25]. Collectively, these findings demonstrate that Nrf2 overexpression promotes neuronal fate shift in endogenous NSCs and facilitates functional recovery in MCAO mice.

### Effects of Nrf2 modulation on lineage fate shift in cultured NSCs

**Fig. 3:**
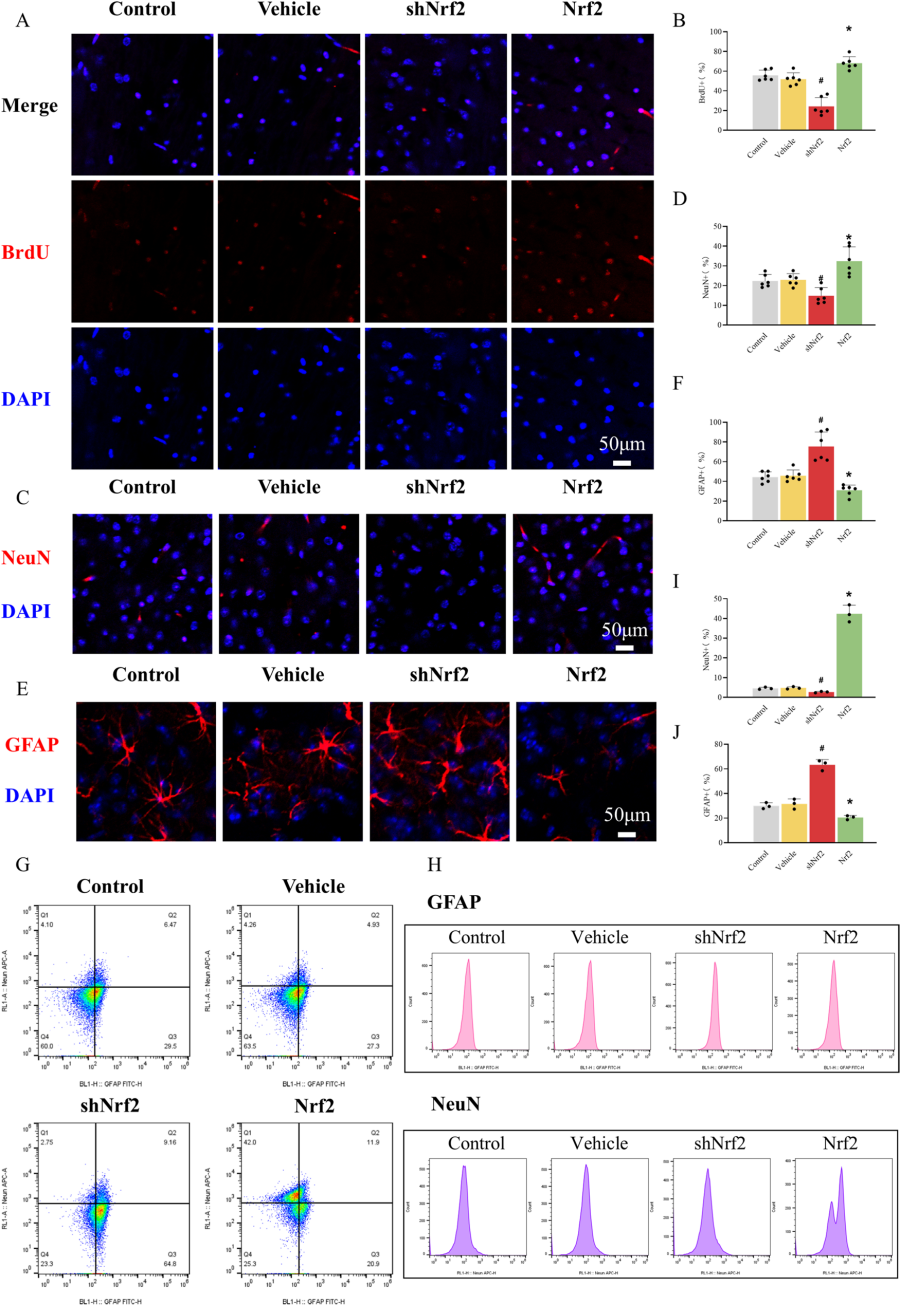
Nrf2 promotes neuronal fate shift of NSCs in vitro. (**A**, **B**) Proliferative potential of NSCs transfected with control vector, scrambled shRNA, Nrf2 shRNA, or Nrf2 overexpression plasmid, was detected using BrdU staining. The histogram showed the percentage of BrdU-positive NSCs among 4 groups. The data are expressed as means ± SD (*n* = 6). ^*^P < 0.05 vs. Control and Vehicle group, ^#^*P* < 0.05 vs. Control and Vehicle group. (**C**, **D**) Differentiation ratio of NSCs to neurons was measured by NeuN immunofluorescence. The histogram showed the percentage of NeuN-positive NSCs among 4 groups. The data are expressed as means ± SD (*n* = 6). ^*^P < 0.05 vs. Control and Vehicle groups, ^#^*P* < 0.05 vs. Control and Vehicle groups. (**E**, **F**) Differentiation ratio of NSCs to glia was measured by GFAP immunofluorescence. The histogram showed the percentage of GFAP-positive NSCs among 4 groups. The data are expressed as means ± SD (*n* = 6). ^*^P < 0.05 vs. Control and Vehicle groups, ^#^*P* < 0.05 vs. Control and Vehicle groups. (**G**, **H**) Representative flow cytometry plots and NeuN or GFAP fluorescence intensity quantification of neural stem cells across four experimental groups. (**I**, **J**) Histogram showing the percentage of NeuN-positive and GFAP-positive cells in four groups. The data are expressed as means ± SD (*n* = 3). ^*^P < 0.05 vs. Control and Vehicle groups, ^#^*P* < 0.05 vs. Control and Vehicle groups

NSCs were transfected with vehicle and AAV-9 to interfere with or overexpress Nrf2, and 48 h later, BrdU staining was performed in all groups. The proportion of BrdU-positive Nrf2-interfered NSCs was significantly lower than that of the control and vehicle groups, whereas the proportion of BrdU-positive Nrf2-overexpressed NSCs was significantly higher than that of the control and vehicle groups (Fig. 3A and B). This indicates that Nrf2 promotes the proliferation of NSCs [43]. Seventy-two hours after infection with AAV-9, approximately 70% of the NSCs had differentiated and adhered to the dishes, and immunofluorescence staining was performed. The results showed that, compared to the control and vehicle group, the proportion of NeuN-positive cells was significantly reduced in the Nrf2-interfered group but increased in the Nrf2-overexpressed group (Fig. 3C and D). In contrast, the proportion of GFAP-positive cells significantly increased in the Nrf2-interfered group and decreased in the Nrf2-overexpressed group (Fig. 3E and F) [44]. Subsequently, we assessed the effects of Nrf2 knockdown or overexpression on NSCs differentiation using flow cytometry [26]. Nrf2 knockdown promoted astrocytic differentiation, evidenced by increased GFAP-positive cell proportion and fluorescence intensity. Conversely, Nrf2 overexpression enhanced neuronal differentiation, indicated by elevated NeuN-positive cell proportion and signal intensity (Fig. 1G-J). These results aligned with immunofluorescence findings. In summary, Nrf2 induces proliferation and promotes a neuronal fate shift in vitro, evidenced by Nrf2 overexpression driving NSCs differentiation toward neurons while suppressing gliogenesis [44].

### Differentiation pattern of transplanted Nrf2-overexpressing NSCs

**Fig. 4:**
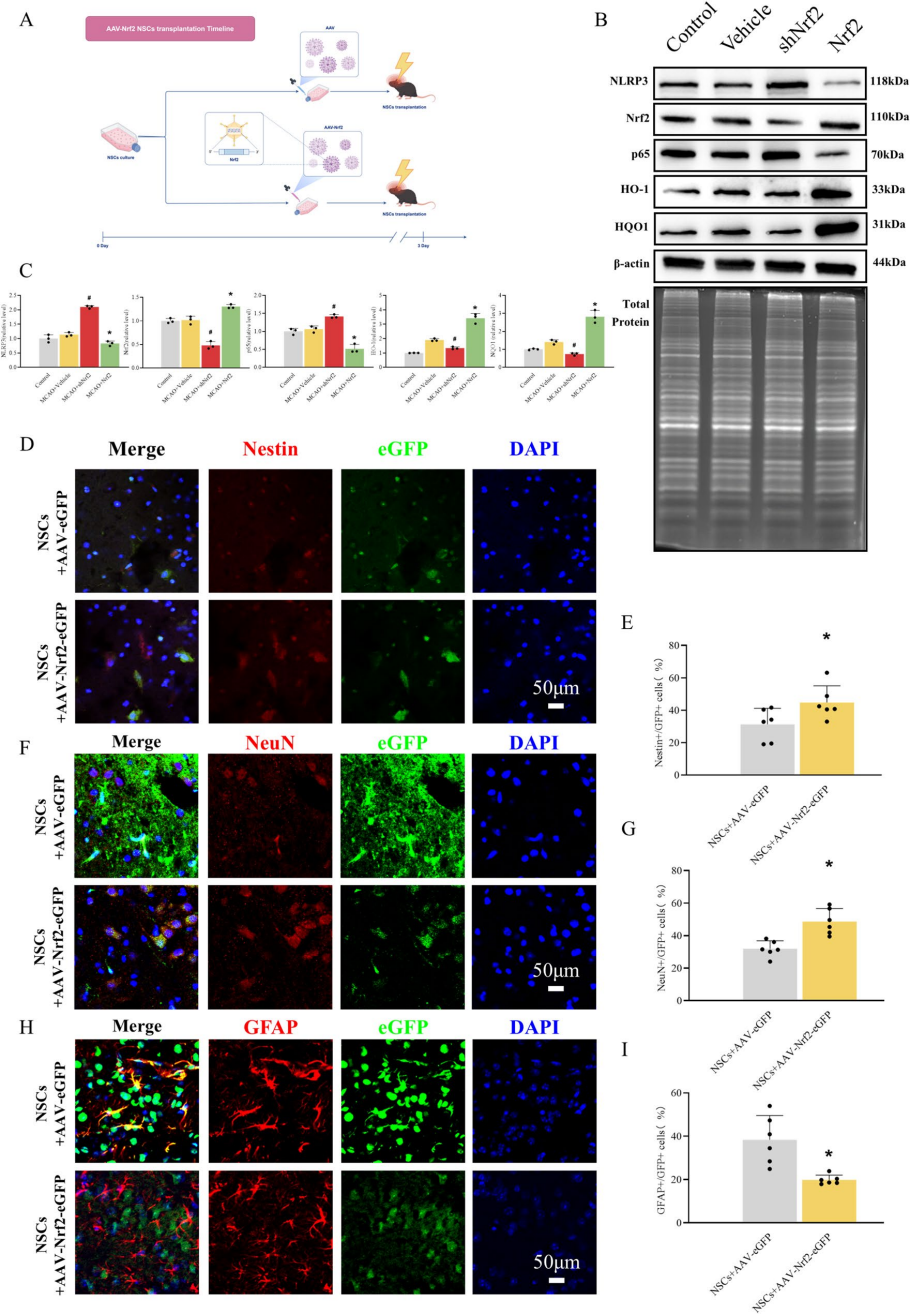
Nrf2-overexpressing NSCs differentiate into neurons post-transplantation. (**A**) Pattern diagram of the transplantation of Nrf2-overexpressing neural stem cells. (**B**, **C**) Western blot analysis of Nrf2, HO-1, HQO1, p65 and NLRP3 expression in brain tissues of each group of mice. The data are expressed as means ± SD (*n* = 3). ^*^P < 0.05 vs. MCAO + Vehicle groups, ^#^*P* < 0.05 vs. MCAO + Vehicle groups. (**D**, **E**) Neuronal fate shift potential was evaluated by Nestin immunofluorescence. The histogram showed the ratio of Nestin+/eGFP + cells. The data are expressed as means ± SD (*n* = 6). ^*^P < 0.05 vs. NSCs + AAV-eGFP group. (**F**-**I**) Differentiative potential of neurons and glia was detected by NeuN and GFAP immunofluorescence. The histograms showed the ratio of NeuN+/eGFP + or GFAP+/eGFP + cells. The data are expressed as means ± SD (*n* = 6). ^*^P < 0.05 vs. NSCs + AAV-eGFP group

Nrf2 or control genes were transfected into NSCs in vitro using AAV-9 labelled with GFP. Each group of NSCs was injected into the lateral ventricle of MCAO mice using a stereotaxic instrument, followed by immunofluorescence staining and Western blotting at 72 h post-transplantation (Fig. 4A). Western blot analysis revealed significantly upregulated expression of Nrf2 and its downstream antioxidant molecules (NQO1 and HO-1) [25] in the transplanted Nrf2-overexpressing NSCs group, whereas the transplantation of Nrf2-knockdown NSCs resulted in markedly reduced expression of these molecules. Concurrently, the expression of inflammatory mediators p65 (a NF-κB subunit) [45] and NLRP3 [46] was suppressed in the Nrf2-overexpressing NSCs transplantation group, whereas their levels were upregulated in the Nrf2-knockdown NSCs group. (Figure 4B and C). Importantly, Nrf2 overexpression increased the proportion of Nestin-positive(Fig. 4D and E) and NeuN-positive cells(Fig. 4F and G), but reduced the proportion of GFAP-positive cells among GFP-positive cells in the MCAO brain (Fig. 4H and I) [14]. In summary, transplantation of Nrf2-overexpressing NSCs into MCAO mice promotes their neuronal fate shift and suppresses gliogenesis.

### Comprehensive assessment of Nrf2-overexpressing NSCs therapy on pathology and function

**Fig. 5:**
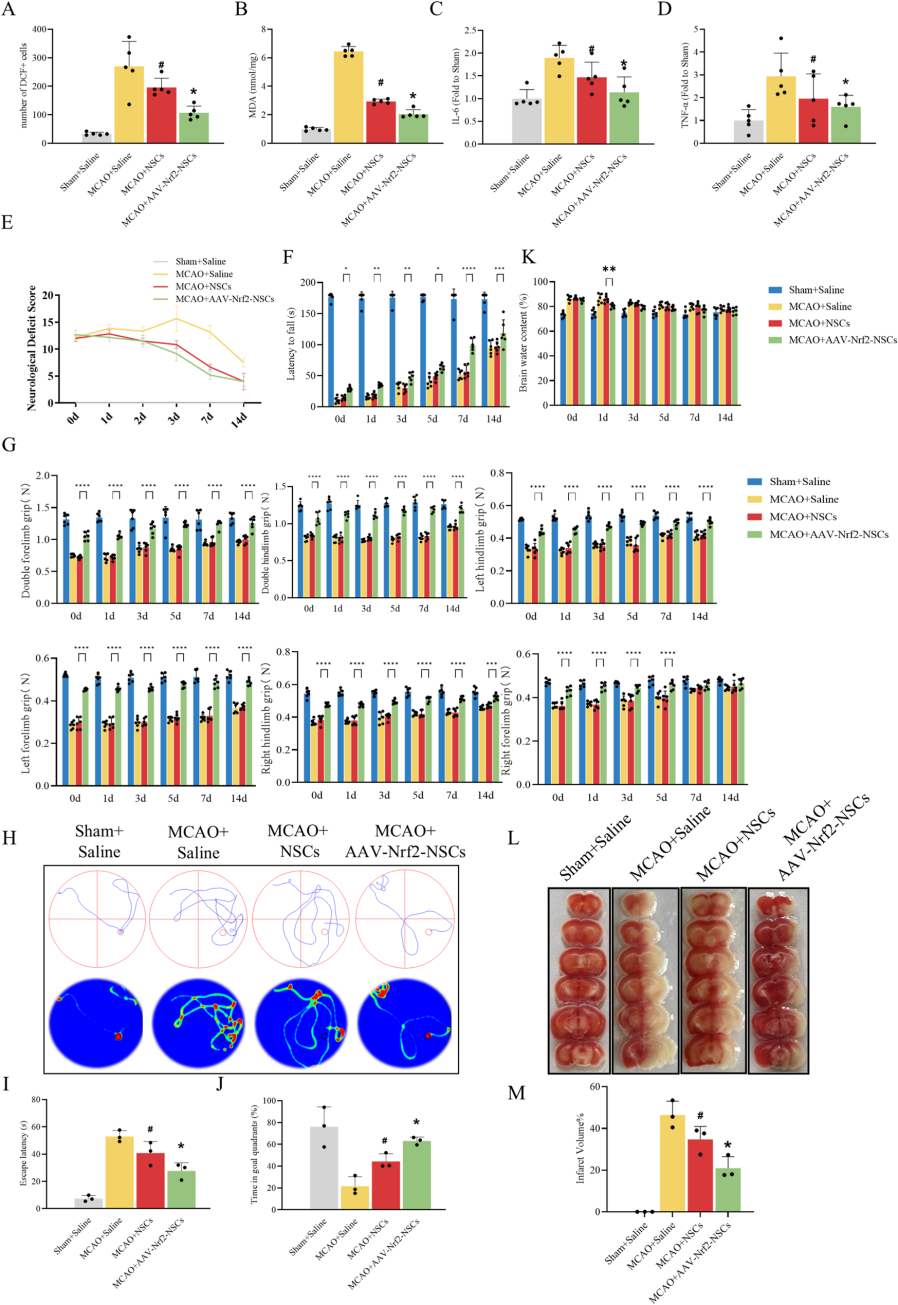
Transplantation of Nrf2-NSCs attenuates damage and improve recovery. (**A**, **B**) Oxidative level was valued by DCF staining and MDA assay. The histograms showed the number of DCF-positive cells and MDA levels. (**C**, **D**) The histograms showed the levels of inflammatory factor IL-6 and TNF-α, measured with ELISA kits. The data are expressed as means ± SD (*n* = 5). ^*^P < 0.05 vs. MCAO + NSCs group, ^#^*P* < 0.05 vs. MCAO + Saline group. (**E**) The curve showed the neurological deficit scores of each group from day 0 to day 14 after MCAO. (**F**) Histogram showing the latency to fall in the rotarod test across four experimental groups. (**G**) Histogram displaying grip strength test results across four experimental groups. The data are expressed as means ± SD (*n* = 6). ^*^P < 0.05, ^**^P < 0.01, ^***^P < 0.001, ^****^P < 0.0001. (**H**-**J**) Representative swimming trajectories of MWM test, bar chart showing escape latency and time spent in the target quadrant. The data are expressed as means ± SD (*n* = 3). ^*^P < 0.05 vs. MCAO + NSCs group, ^#^*P* < 0.05 vs. MCAO + Saline group. (**K**) Histogram quantifying brain water content (%) across four experimental groups. The data are expressed as means ± SD (*n* = 6), ^**^P < 0.01. (**L**, **M**) Representative TTC staining and quantitative analysis of the infarct volume in each group. The data are expressed as means ± SD (*n* = 3). ^*^P < 0.05 vs. MCAO + NSCs group, ^#^*P* < 0.05 vs. MCAO + Saline group

Brain tissues were harvested 7 days post-transplantation of NSCs for subsequent analyses. DCFH-DA staining showed that after MCAO modelling, NSCs injection decreased the proportion of DCF-positive cells in the MCAO brain, and this decrease was more significant when mice were injected with Nrf2-overexpressed NSCs (Fig. 5A) [42]. Simultaneously, MDA, IL-6, and TNF-α levels were assessed using unfixed tissue. It was found that MCAO increased the level of MDA, IL-6, and TNF-α, and that was reduced by NSCs injections [47]. Significantly, when compared with NSCs, injection of Nrf2-overexpressed NSCs downregulated the MDA, IL-6, and TNF-α levels (Fig. 5B-D). These results indicate that Nrf2 overexpression enhances the anti-inflammatory and anti-oxidative effects of NSCs in the MCAO brain. Next, each group of mice was assessed using the Clark neurological deficits score, and the results showed that the NSCs injections ameliorated the neurological deficits in MCAO mice, with a significant decrease in the neurological deficit score curve [48], which was more significant when the NSCs were overexpressed with Nrf2, with a greater decrease in the neurological deficit score curve (Fig. 5E). Concurrently, supplementary behavioural assessments were conducted as detailed below. The rotarod test revealed that MCAO mice transplanted with NSCs exhibited prolonged latency to fall, while those receiving Nrf2-overexpressing NSCs showed a more pronounced prolongation of fall latency (Fig. 5F). Grip strength test demonstrated enhanced forelimb/hindlimb strength in MCAO mice transplanted with NSCs, while those receiving Nrf2-overexpressing NSCs exhibited more pronounced enhancement of grip strength (Fig. 5G). Morris water maze results demonstrated similar therapeutic effects: NSCs transplantation ameliorated prolonged escape latency and reduced target quadrant duration in MCAO mice [49], with even more significant improvements observed in mice receiving Nrf2-overexpressing NSCs (Fig. 5H-J). Notably, cerebral water content measurements at designated time points revealed that transplantation of Nrf2-overexpressing NSCs provided superior therapeutic efficacy against cerebral oedema compared to NSCs transplantation exclusively on day 1 post-MCAO, with no significant difference observed at later time points. This transient enhancement may be attributable to synergistic anti-inflammatory effects of NSCs and the intrinsic reparative capacity of the mice (Fig. 5K). Finally, TTC staining was utilized to assess the neuroprotective effect of Nrf2-overexpressing NSCs transplantation on cerebral infarction volume in MCAO mice. Quantitative analysis demonstrated reduced infarction volume in the NSCs transplantation group (34.68% ± 6.27%) compared to the MCAO group (46.51% ± 6.51%) [16], with a more pronounced reduction observed in the Nrf2-overexpressing NSCs transplantation group (20.97% ± 5.44%) (Fig. 5L and M). Collectively, these findings demonstrate that Nrf2 overexpression potentiates the therapeutic efficacy of NSCs transplantation, ameliorates neuroinflammation and oxidative stress, and rescues neurological function in MCAO mice [50].

### Synergistic therapeutic outcomes of combined PTE and Nrf2-overexpressing NSCs therapy

**Fig. 6:**
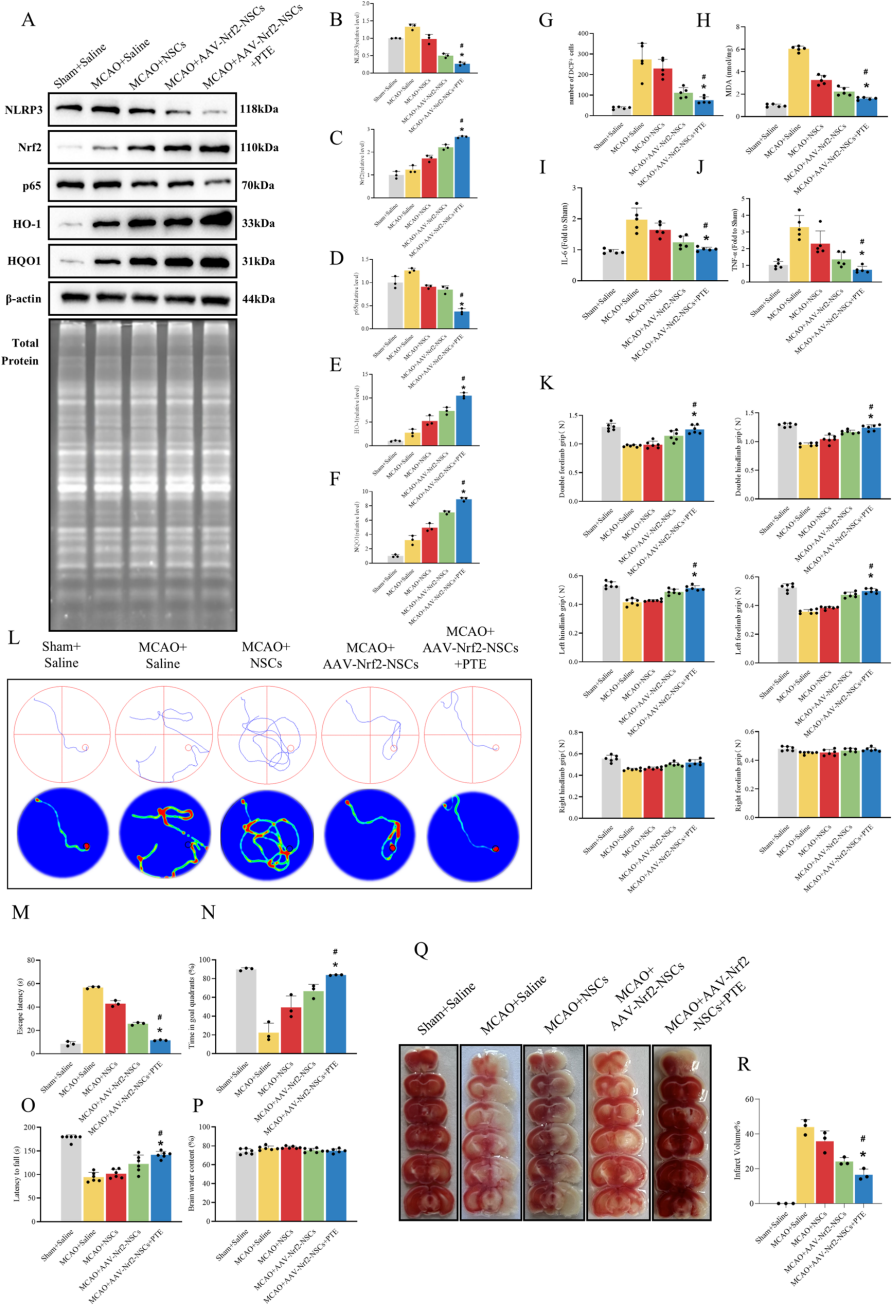
Pterostilbene synergizes with Nrf2-NSCs for enhanced therapy. (**A**-**F**) Western blot analysis of Nrf2, HO-1, HQO1, p65 and NLRP3 expression in brain tissues of each group of mice. The data are expressed as means ± SD (*n* = 3). ^*^P < 0.05 vs. MCAO + AAV-Nrf2-NSCs group, ^#^*P* < 0.05 vs. MCAO + Saline group. (**G**, **H**) Oxidative level was valued by DCF staining and MDA assay. The histograms showed the number of DCF-positive cells and MDA levels. (**I**, **J**) The histograms showed the levels of inflammatory factor IL-6 and TNF-α, measured with ELISA kits. The data are expressed as means ± SD (*n* = 5). ^*^P < 0.05 vs. MCAO + AAV-Nrf2-NSCs group, ^#^*P* < 0.05 vs. MCAO + Saline group. (**K**) Histogram displaying grip strength test results across five experimental groups. The data are expressed as means ± SD (*n* = 6). ^*^P < 0.05 vs. MCAO + AAV-Nrf2-NSCs group, ^#^*P* < 0.05 vs. MCAO + Saline group. (**L**-**N**) Representative swimming trajectories of MWM test, bar chart showing escape latency and time spent in the target quadrant. The data are expressed as means ± SD (*n* = 3). ^*^P < 0.05 vs. MCAO + AAV-Nrf2-NSCs group, ^#^*P* < 0.05 vs. MCAO + Saline group. (**O**) Histogram showing the latency to fall in the rotarod test across five experimental groups. The data are expressed as means ± SD (*n* = 6). ^*^P < 0.05 vs. MCAO + AAV-Nrf2-NSCs group, ^#^*P* < 0.05 vs. MCAO + Saline group. (**P**) Histogram quantifying brain water content (%) across five experimental groups. The data are expressed as means ± SD (*n* = 6). (**Q**, **R**) Representative TTC staining and quantitative analysis of the infarct volume in each group. The data are expressed as means ± SD (*n* = 6). ^*^P < 0.05 vs. MCAO + AAV-Nrf2-NSCs group, ^#^*P* < 0.05 vs. MCAO + Saline group

**Fig. 7:**
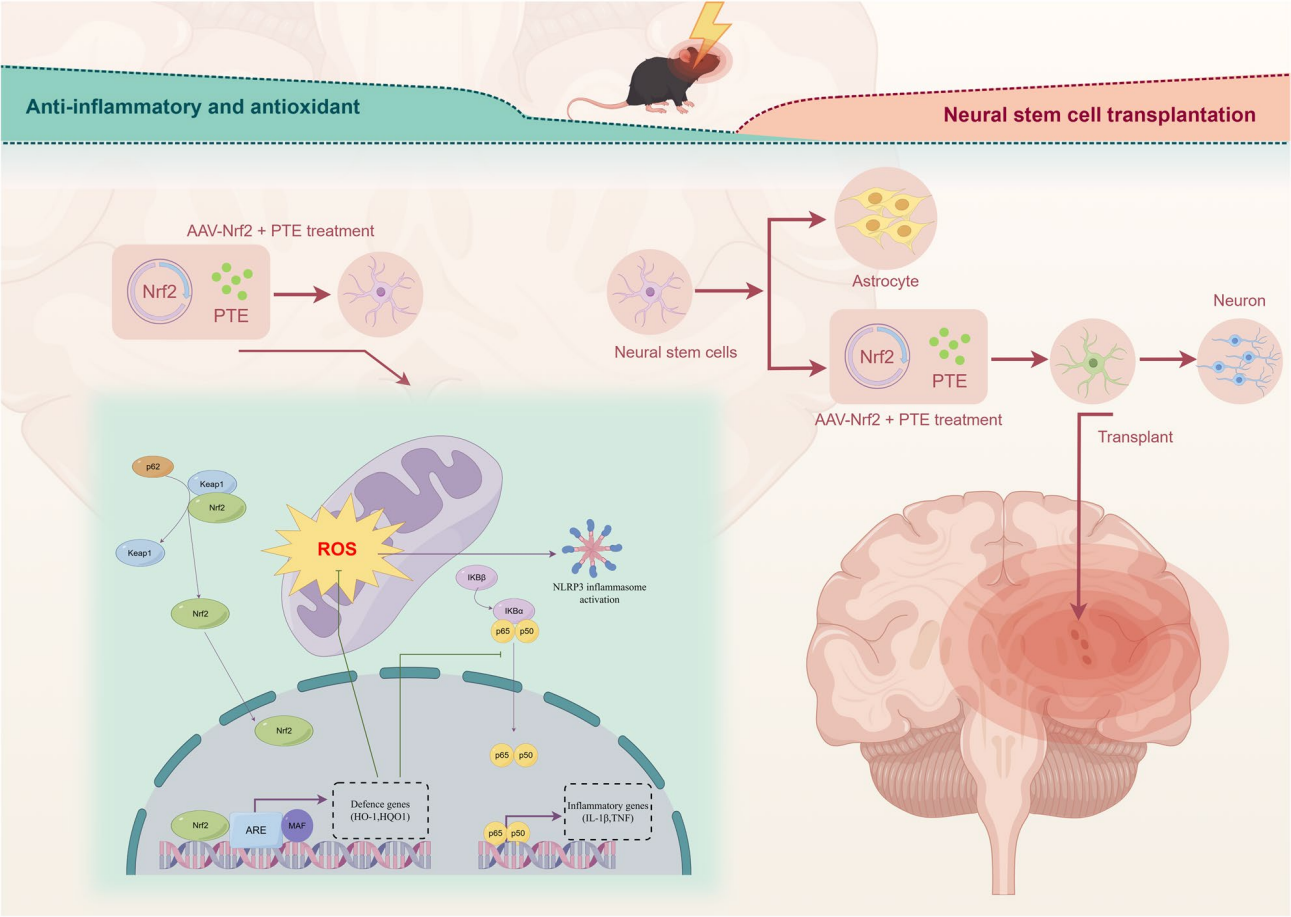
Mechanism of Nrf2 in NSCs fate shift post-ischemic stroke

To further investigate the role of Nrf2 in NSCs transplantation for MCAO mice, we administered PTE, a natural Nrf2 agonist, post-transplantation [51]. Brain tissues were harvested 14 days after PTE injection for Western blotting, ELISA, histological analyses, behavioural assessments and other experiments. Western blot analysis demonstrated that the combined regimen of Nrf2-overexpressing NSCs transplantation and PTE injection exerted synergistic effects, significantly upregulating and activating the Nrf2 signalling pathway(evidenced by elevated expression of Nrf2, NQO1 and HO-1) [52]while suppressing p65 [23] and NLRP3 expression (Fig. 6A-F) [51]. DCFH-DA staining further validated the synergistic interaction between interventions. Compared to transplantation of Nrf2-overexpressing NSCs alone, the combined regimen with PTE treatment dramatically reduced the proportion of DCF-positive cells in MCAO brains (Fig. 6G) [23]. Concurrently, assessment of MDA, IL-6, and TNF-α levels revealed a similar synergistic pattern. Administration of PTE further potentiated the capacity of Nrf2-overexpressing NSCs transplantation to downregulate these biomarkers (Fig. 6H-J) [53]. To evaluate long-term neurological outcomes, behavioural assessments and histological analyses were performed at 14 days post-intervention. The rotarod test demonstrated that mice receiving the combined regimen of PTE and Nrf2-overexpressing NSCs transplantation exhibited a significantly greater prolongation of fall latency compared to those treated with Nrf2-overexpressing NSCs transplantation alone (Fig. 6O). Consistent with these findings, grip strength test revealed a significantly greater enhancement of forelimb/hindlimb strength in mice receiving the combined regimen of PTE and Nrf2-overexpressing NSCs transplantation (Fig. 5K). Notably, consistent with previous findings, ipsilateral forelimb weakness was observed in MCAO mice but showed complete recovery by day 14, and no significant difference in ipsilateral forelimb/hindlimb strength was observed between the combination PTE therapy group and the Nrf2-overexpressing NSCs transplantation group. Morris water maze results demonstrated that the combined regimen of PTE and Nrf2-overexpressing NSCs transplantation augmented the previously described improvements observed with Nrf2-overexpressing NSCs transplantation alone, further ameliorating prolonged escape latency and increase target quadrant duration in MCAO mice (Fig. 6L-N) [22]. Notably, cerebral water content measurements at 14 days revealed no significant difference between the two interventional groups (Fig. 6P). Finally, TTC staining was employed to evaluate the neuroprotective effects of both interventional approaches on cerebral infarction volume in MCAO mice. Quantitative analysis revealed a significantly greater reduction in infarction volume with the combined regimen of PTE and Nrf2-overexpressing NSCs transplantation (16.59% ± 3.22%) compared to Nrf2-overexpressing NSCs transplantation alone (24.17% ± 2.22%) (Fig. 6Q and R) [22]. Collectively, these findings demonstrate that the combined regimen of PTE and Nrf2-overexpressing NSCs transplantation activates the Nrf2/HO-1 pathway, suppresses p65 and NLRP3 expression, synergistically augments anti-inflammatory and antioxidant effects, and rescues neurofunctional outcomes in MCAO mice (as mechanistically summarized in Fig. 7).

## Discussion

Stem cell-based therapies have been tested in various experimental models and patients [6–8]. Although studies have reported that stereotaxic injections of NSCs can improve neurological function in stroke patients [54] and induce no adverse events [6], many studies have found that few NSCs differentiate and properly migrate into the damaged area, integrating into host neuronal circuits [8, 54]. In addition, NSCs may not survive and maintain stable proliferation and differentiation because of damage from long-term in vitro replication and ischaemic environments [7, 8, 10]. Multiple pathological events, including excitotoxicity, oxidative stress, and inflammatory responses, are known to cause cell death after a stroke [1], leading to impaired survival, proliferation, and multi-lineage differentiation of endogenous NSCs [5, 9, 11, 12]. Studies have suggested that the survival and neuronal differentiation of NSCs grafts are limited when transplanted after ischaemic stroke [8]. In this context, our single-cell sequencing analysis provides mechanistic insight into this limitation, revealing that NSCs in the MCAO brain exhibit a transcriptional bias towards glial lineage commitment, marked by upregulation of Stat3, Smad5, Nfia, and Sox9. This finding is consistent with the established role of NSCs in supporting central nervous system repair through glial cell production [31, 33], suggesting that the ischemic environment actively skews their fate toward a supportive rather than a neuronal-replacement program.

Given that this hostile milieu is characterized by oxidative stress and inflammation [1], we reasoned that enhancing endogenous antioxidant defences could be a strategy to counteract the glial bias and promote a neurogenic program. We therefore targeted Nrf2, hypothesizing that its activation could reprogram NSCs fate within the ischemic brain. Nrf2 is a pivotal antioxidant factor that regulates multiple antioxidant factors and plays an important role in maintaining redox balance. Recently, it has been suggested that Nrf2 protects NSCs against aging by promoting NSCs proliferation, self-renewal, neurogenesis, and migration [15]. However, its specific role in determining NSCs fate within the pathological context of cerebral ischemia, and crucially, its potential to enhance the therapeutic efficacy of exogenous NSCs transplantation, remained unexplored. To address this, we systematically investigated the effects of Nrf2 gain- and loss-of-function on NSCs biology, both in vitro and within the ischemic mouse brain, and further evaluated the therapeutic potential of Nrf2-overexpressing NSCs grafts. Our data establish Nrf2 as a decisive regulator of NSCs fate under ischemic conditions. The gain- and loss-of-function experiments in vitro confirm that Nrf2 activity is both necessary and sufficient to drive NSCs proliferation and neuronal lineage commitment. This intrinsic role of Nrf2 in directing NSCs differentiation aligns with its reported pro-neurogenic function in models of aging [15] and neurodegeneration [14], extending its importance to the acute phase of ischemic injury. Crucially, our in vivo findings demonstrate that enhancing Nrf2 signalling within the endogenous NSCs niche not only recapitulates these pro-neurogenic effects but also potently counteracts the hallmark pathologies of the post-ischemic brain—namely, oxidative stress and neuroinflammation. This dual action suggests that Nrf2 activation creates a more permissive microenvironment for neurogenesis by mitigating the very factors that originally skewed NSCs fate toward gliogenesis. This “dual-action” mechanism extends the prevailing view of Nrf2 in stroke, which has largely focused on its direct cytoprotective effects in mature neurons and astrocytes [25, 40]. Our work positions Nrf2 upstream as a fate-determining regulator within the NSCs population itself, thereby enabling a more fundamental repair by increasing the pool of neuron-committed precursors while simultaneously improving their survival milieu. This concept is strongly supported by prior evidence that Nrf2 controls the fate choice between neurogenesis and gliogenesis in the hippocampal niche [44].

Building on the role of Nrf2 in endogenous repair, we translated this knowledge into a therapeutic strategy by engineering NSCs to stably overexpress Nrf2. These modified grafts possessed intrinsically enhanced resilience, likely due to their fortified antioxidant defences. This approach aligns with the concept of preconditioning or engineering stem cells to better withstand the harsh transplantation milieu [7, 8], and indeed, transplantation of Nrf2-overexpressing NSCs led to significant functional recovery, confirming their superior therapeutic potency. While other preconditioning strategies, such as cytokine exposure, aim to boost general cell survival and paracrine function [8, 32], our Nrf2-engineering strategy is distinctive in its dual targeting: it concurrently enhances graft resilience (antioxidant defence) and actively biases their differentiation fate (pro-neurogenic programming). This may address the two most critical limitations—poor survival and limited neuronal differentiation—in a more integrated manner.

The therapeutic benefits of Nrf2-overexpressing NSCs grafts likely stem from a potentiation of the known bystander mechanisms of stem cell therapy. While NSCs are recognized to promote repair through trophic support and immunomodulation [5, 7, 8], our findings suggest that elevating Nrf2 within grafts amplifies these paracrine capacities, particularly the anti-inflammatory and antioxidant effects. This enhancement, coupled with the observed increase in graft-derived neuronal differentiation, indicates that Nrf2 engineering shifts the therapeutic profile from predominantly modulatory to include a stronger element of cell replacement. Consequently, these fortified grafts more effectively remodel the ischemic niche, not only by directly supplying resilient, neuron-committed cells but also by creating a more supportive milieu through amplified neuroprotection. This mechanistic synergy between intrinsic cell fate reprogramming and augmented paracrine action underpins their superior neurological prognosis. Furthermore, the synergistic effect achieved by combining Nrf2-overexpressing NSCs with the pharmacological activator PTE underscores a promising paradigm: concurrently fortifying the transplanted cells and modulating the host environment can amplify therapeutic outcomes beyond what either approach achieves alone. This “dual-pathway activation” paradigm differs from most reported combination therapies for stroke, which often pair stem cells with broad-spectrum anti-inflammatory agents (e.g., minocycline) or neurotrophic factors [47]. Our approach ensures that both the host environment and the graft are modulated through a coherent molecular axis (Nrf2), potentially creating a more specific and potent synergistic loop. The efficacy of PTE, a natural Nrf2 pathway activator used in our study, in protecting the neurovascular unit further supports the rationality of this targeted combination [53]. While the precise long-term integration and functional connectivity of the Nrf2-induced neurons warrant further investigation, our results robustly validate Nrf2 as a pivotal target for enhancing neural repair after ischemic stroke.

Nevertheless, this study has certain limitations. In the immunofluorescence analysis of mouse brain tissue sections presented in Result 3, we cannot definitively conclude whether the observed GFAP-positive cells originate from the differentiation of NSCs or from the proliferation of reactive astrocytes [55, 56]. This ambiguity arises because NSCs lose their stem cell markers upon differentiation into mature cell types [57–59], preventing their identification via dual-labelling strategies for tracing. Furthermore, reactive astrogliosis in pathological conditions is also characterized by upregulated GFAP expression [60, 61], complicating the discrimination of the cellular source of GFAP-positive cells. To partially address this issue, we employed flow cytometry analysis of in vitro-cultured NSCs and performed immunofluorescence staining on brain tissues from mice that had received transplants of eGFP-labelled NSCs, which provides lineage-tracing capability. Collectively, these complementary lines of evidence indicate that the increase in GFAP-positive cells is at least partially attributable to the differentiation of NSCs into astrocytes, rather than solely being a result of reactive astrocytic proliferation.

## Supplementary Information

Below is the link to the electronic supplementary material.

Supplementary Material 1

Supplementary Material 2

Supplementary Material 3

Supplementary Material 4

## Data Availability

The raw data supporting the conclusions of this article will be made available by the authors, without undue reservation.

## Abbreviations

AAV-9: Adeno-associated virus vectors-9
BrdU: Bromodeoxyuridine (5-Bromo-2’-deoxyuridine)
BSA: Bovine serum albumin
CCK-8: Cell Counting Kit-8
CIS: Cerebral ischaemic stroke
DCF: Dichlorofluorescein (from DCFH-DA, 2′,7′-dichlorodihydrofluorescein diacetate)
Dcx: Doublecortin
DEGs: Differentially expressed genes
eGFP: Enhanced green fluorescent protein
ELISA: Enzyme-Linked Immunosorbent Assay
GEO: Gene Expression Omnibus
GFAP: Glial fibrillary acidic protein
GO: Gene Ontology
HO-1: Heme oxygenase-1
IL-6: Interleukin-6
KEGG: Kyoto Encyclopedia of Genes and Genomes
MCAO: Middle cerebral artery occlusion
MDA: Malondialdehyde
MOI: Multiplicity of infection
MWM: Morris water maze
NF-κB: Nuclear factor kappa-light-chain-enhancer of activated B cells (Nuclear factor kappa-B)
NLRP3: Nucleotide-binding oligomerization domain (NOD)-like receptor family, pyrin domain containing 3
NQO1: NAD(P)H quinone oxidoreductase 1
Nrf2: Nuclear factor erythroid 2-related factor 2
NSCs: Neural stem cells
NeuN: Neuron-specific nuclear protein
p65: Transcription factor p65 (Nuclear Factor NF-kappa-B p65 subunit)
PTE: Pterostilbene
PVDF: Polyvinylidene fluoride
ROS: Reactive oxygen species
scRNA-seq: Single-cell RNA sequencing
SD: Standard deviation
shRNA: Short hairpin RNA
TNF-α: Tumour necrosis factor-alpha
t-SNE: t-Distributed Stochastic Neighbour Embedding
TTC: 2,3,5-Triphenyltetrazolium Chloride
