# Supplementary material for: Nrf2 overexpression reprograms neural stem cell fate: promoting neuronal differentiation and functional recovery post-ischemic stroke via suppression of the ROS/NF-κB axis
Source: J Transl Med. 2026 Jan 14;24:169. doi: 10.1186/s12967-025-07675-w (PMC12888667; doi:10.1186/s12967-025-07675-w)

Fig.2O

Nestin:

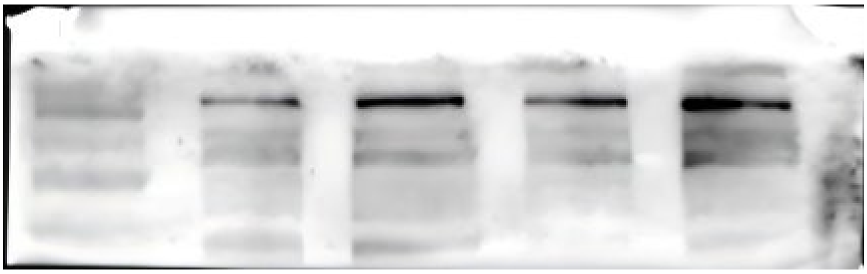

DCX:

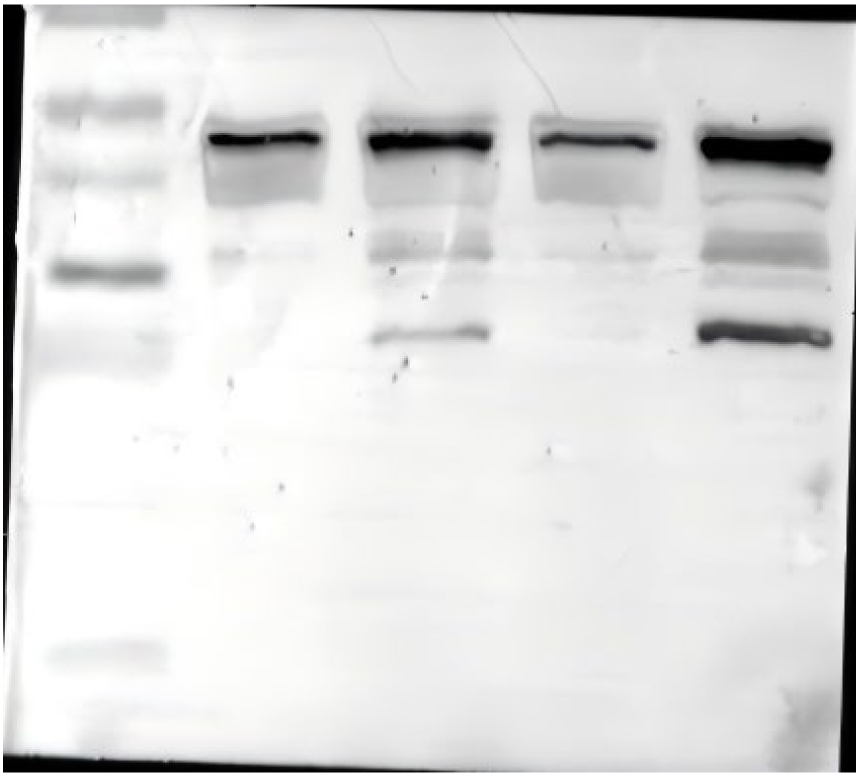

GAPDH:

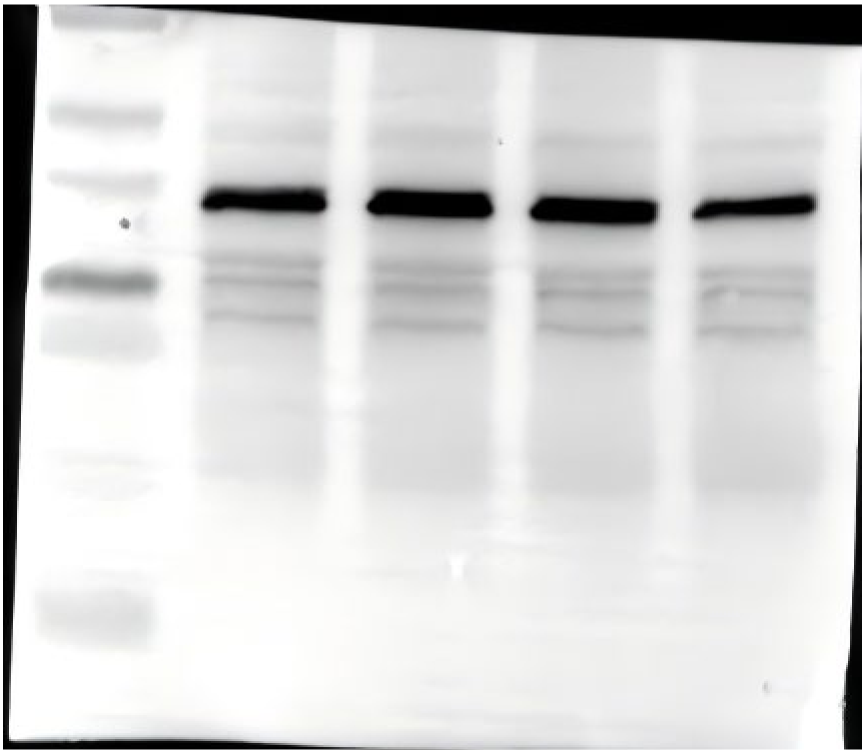

Total protein：

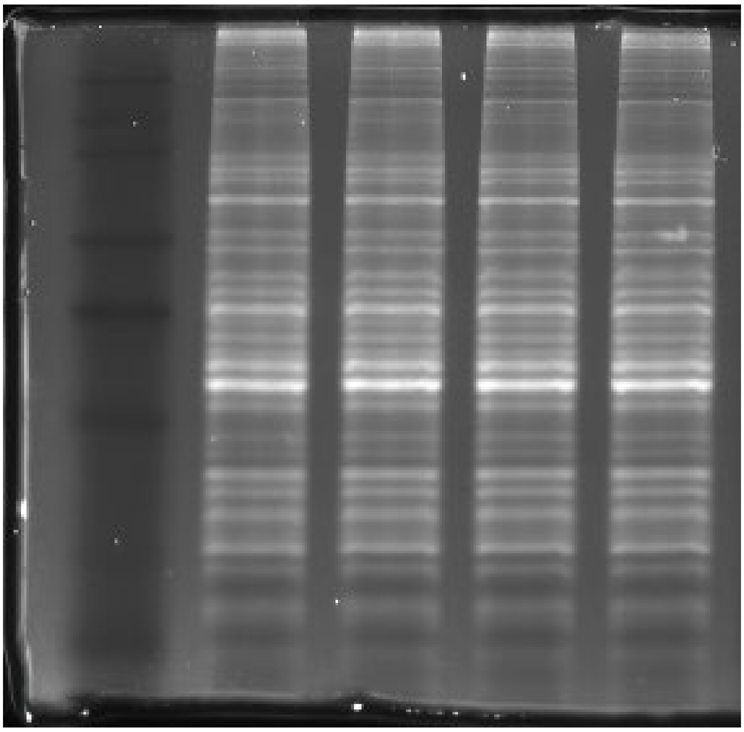

PVDF membrane:

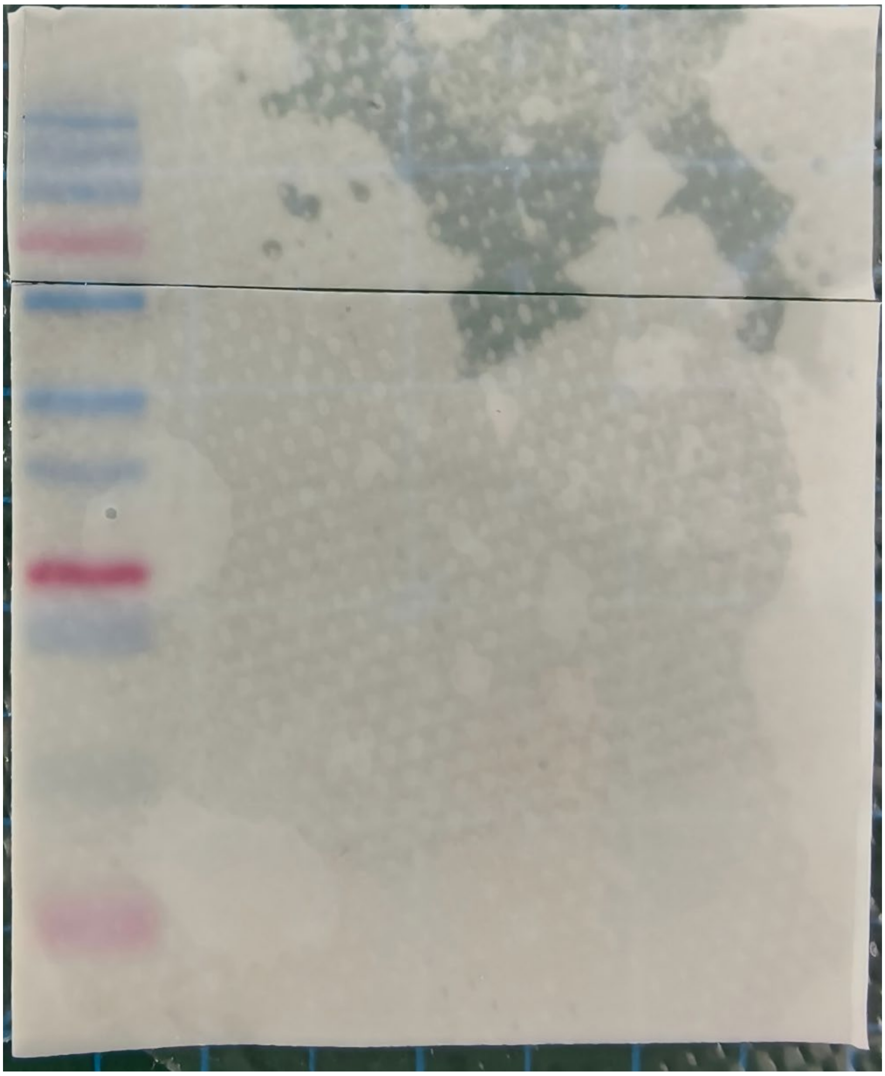

Fig.4B
NLRP3:

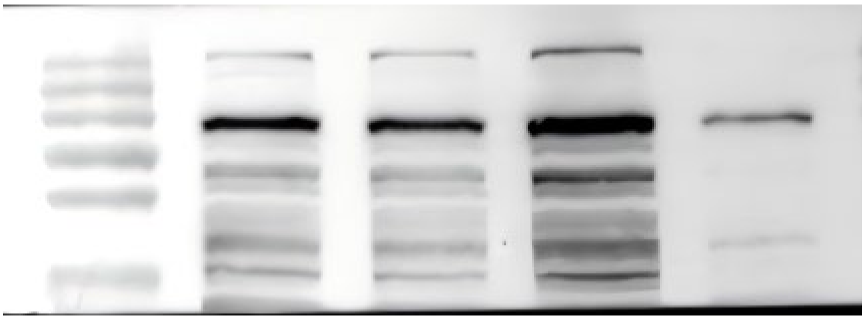

Nrf2:

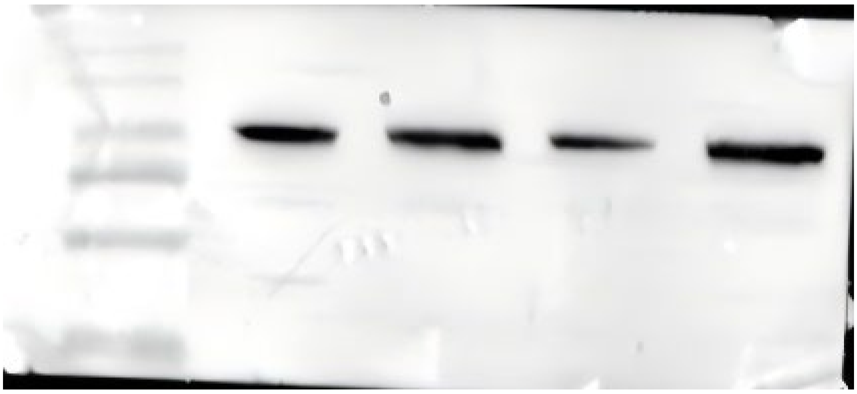

P65:

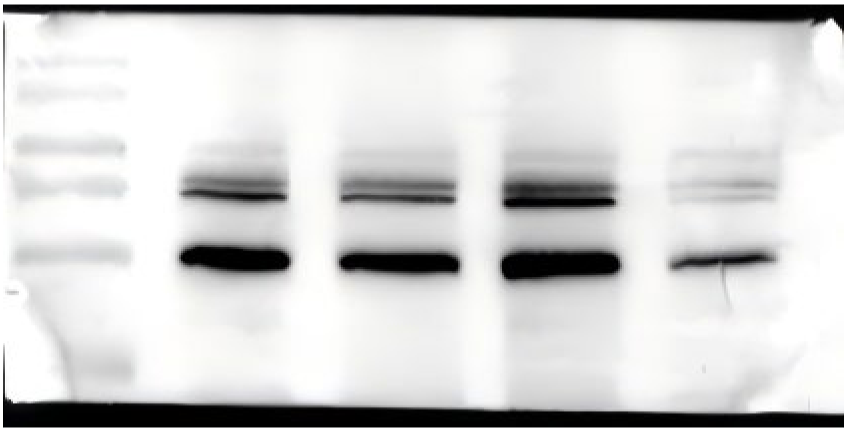

HO-1:

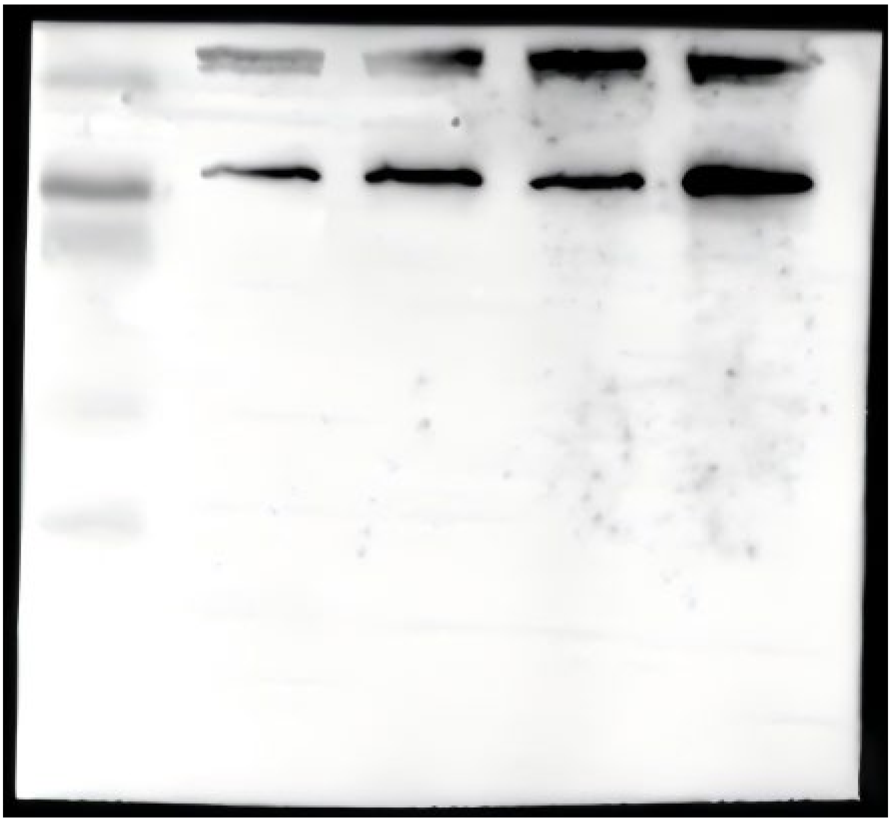

HQO1:

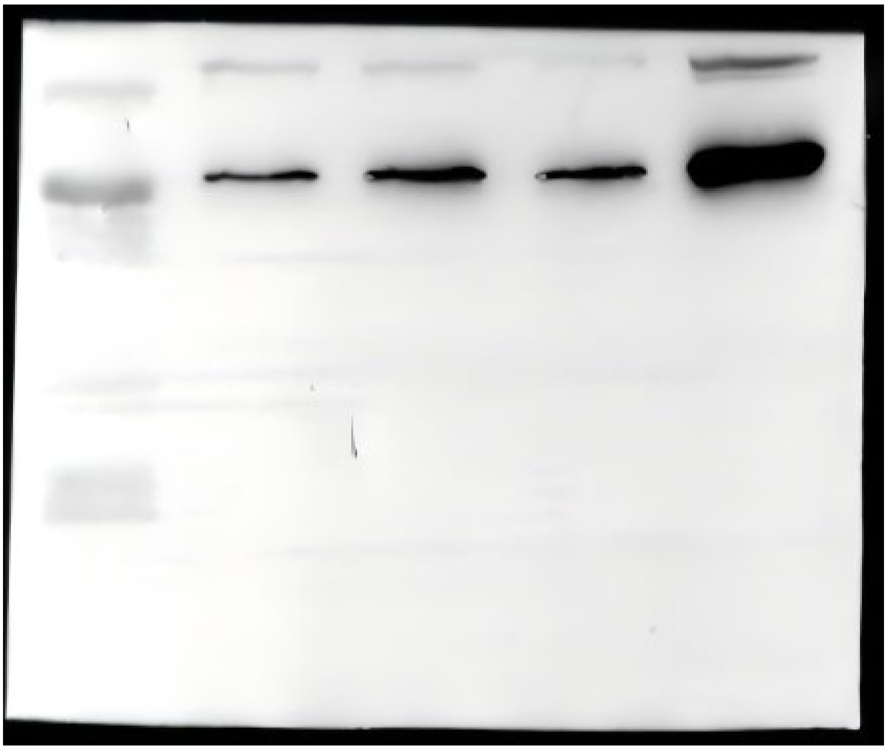

β-actin：

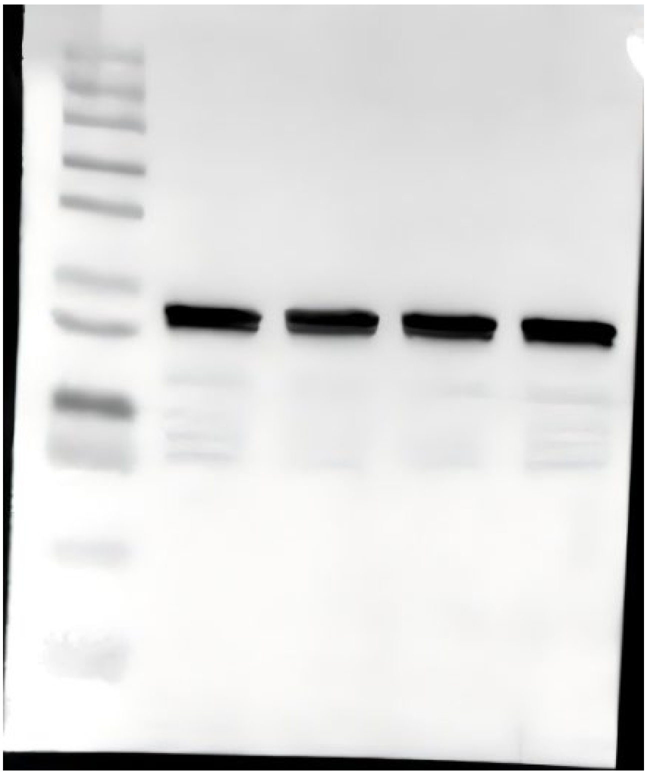

Total protein：

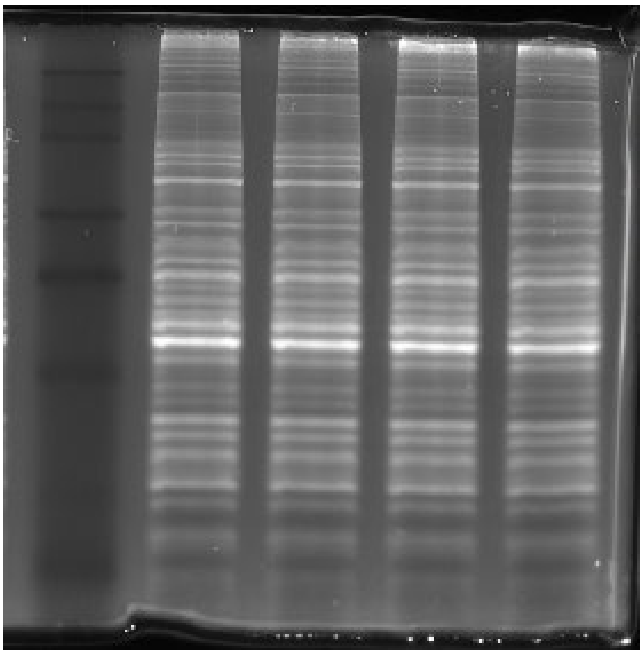

PVDF membrane:

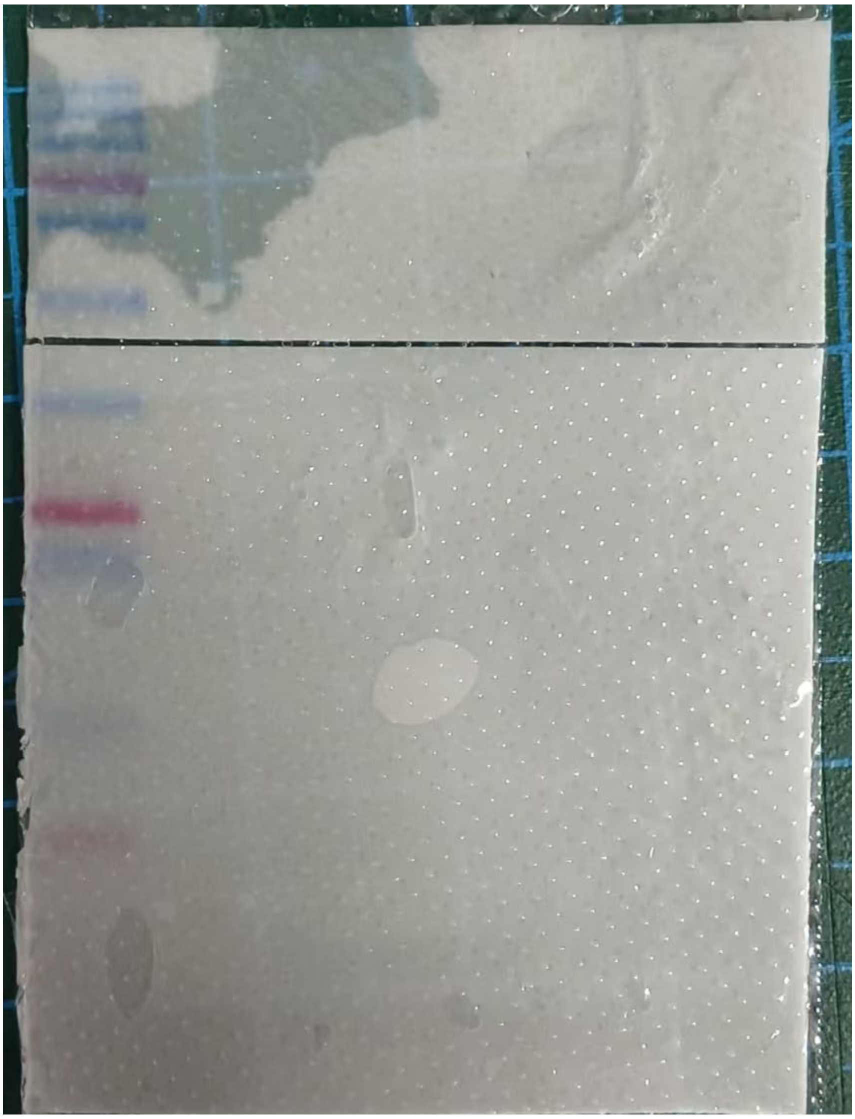

Fig.6A

NLRP3：

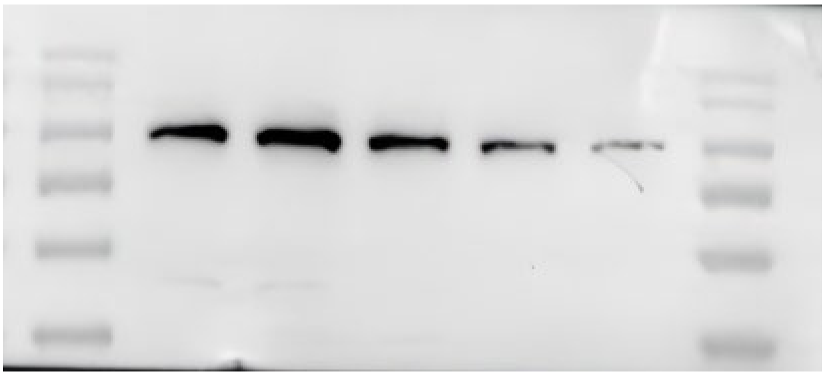

Nrf2：

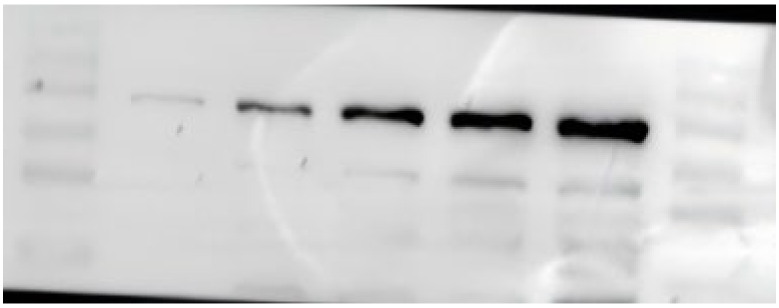

P65：

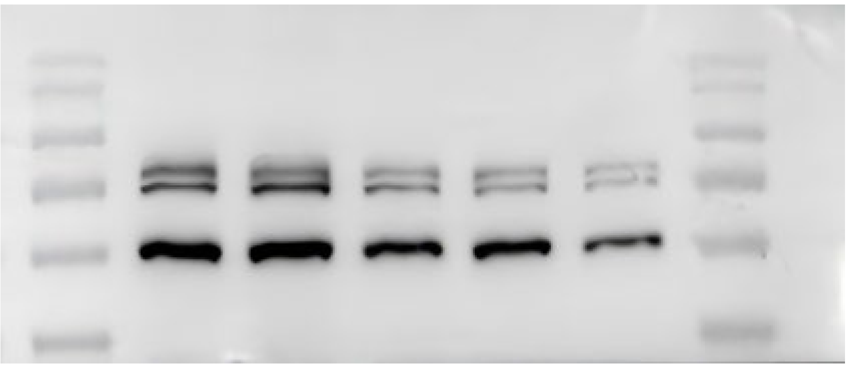

HO-1：

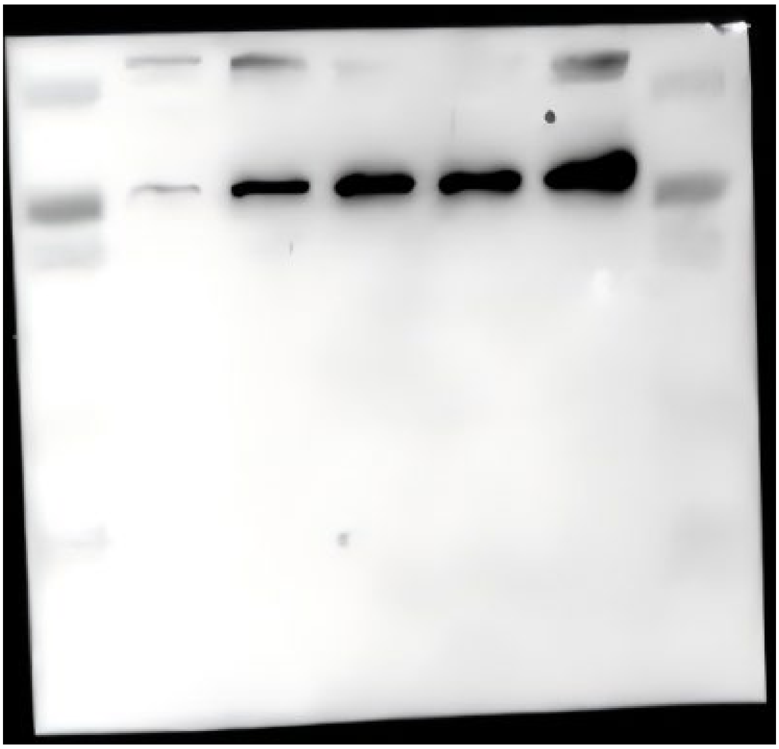

HQO1：

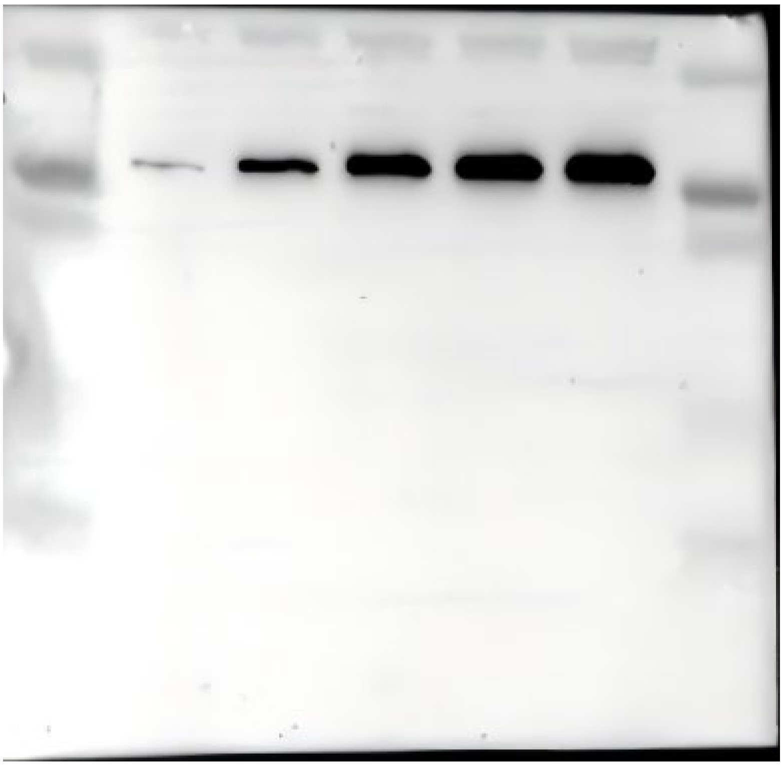

β-actin：

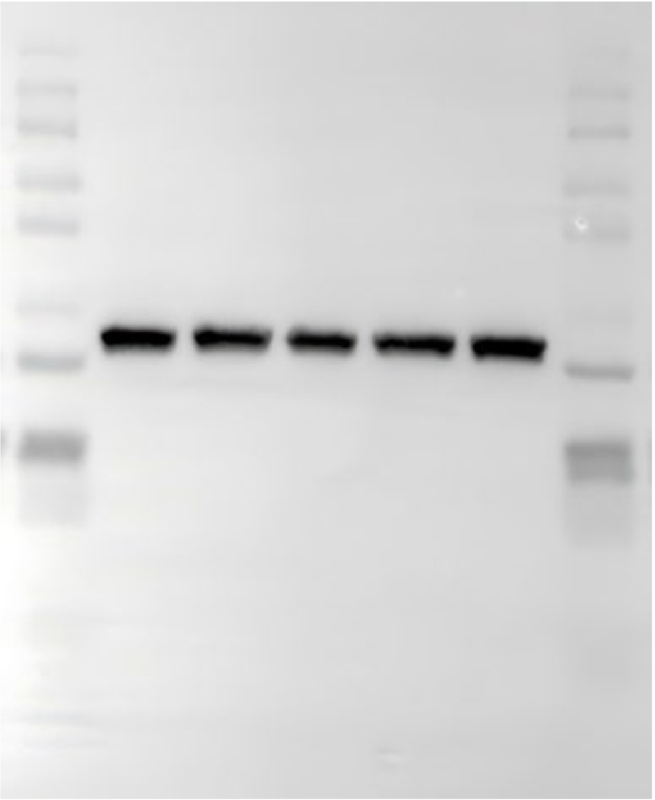

Total protein:

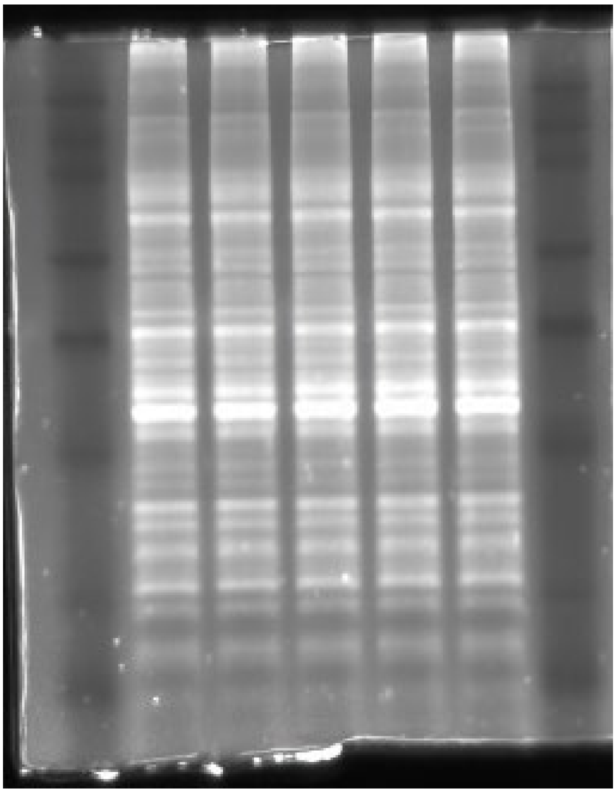

PVDF membrane:

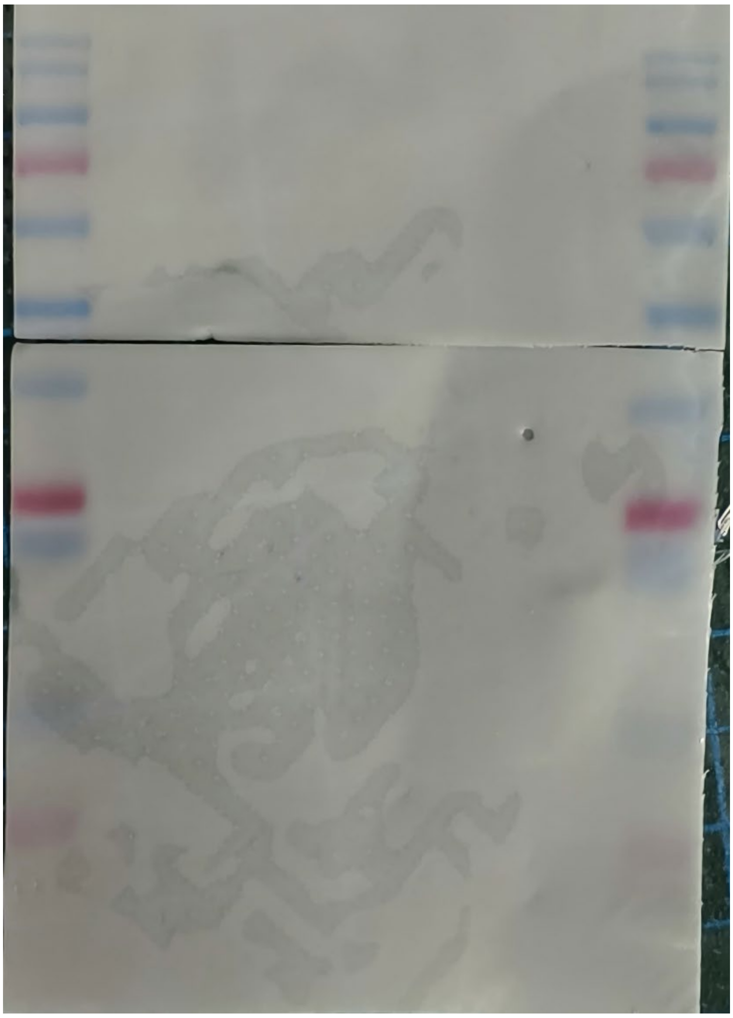

Supplement: Supplementary file 3 — Supplementary Material 3 [file 12967_2025_7675_MOESM3_ESM.pdf]
